# Glycogen Synthase Kinase (GSK) 3β Phosphorylates and Protects Nuclear Myosin 1c from Proteasome-Mediated Degradation to Activate rDNA Transcription in Early G1 Cells

**DOI:** 10.1371/journal.pgen.1004390

**Published:** 2014-06-05

**Authors:** Aishe A. Sarshad, Martin Corcoran, Bader Al-Muzzaini, Laura Borgonovo-Brandter, Anne Von Euler, Douglas Lamont, Neus Visa, Piergiorgio Percipalle

**Affiliations:** 1 Department of Cell and Molecular Biology, Karolinska Institute, Stockholm, Sweden; 2 Department of Molecular Biosciences, The Wenner-Gren Institute, Stockholm University, Stockholm, Sweden; 3 FingerPrints Proteomics Facility, College of Life Sciences, University of Dundee, Dundee, United Kingdom; University of Zurich, Switzerland

## Abstract

Nuclear myosin 1c (NM1) mediates RNA polymerase I (pol I) transcription activation and cell cycle progression by facilitating PCAF-mediated H3K9 acetylation, but the molecular mechanism by which NM1 is regulated remains unclear. Here, we report that at early G1 the glycogen synthase kinase (GSK) 3β phosphorylates and stabilizes NM1, allowing for NM1 association with the chromatin. Genomic analysis by ChIP-Seq showed that this mechanism occurs on the rDNA as active GSK3β selectively occupies the gene. ChIP assays and transmission electron microscopy in GSK3β^−/−^ mouse embryonic fibroblasts indicated that at G1 rRNA synthesis is suppressed due to decreased H3K9 acetylation leading to a chromatin state incompatible with transcription. We found that GSK3β directly phosphorylates the endogenous NM1 on a single serine residue (Ser-1020) located within the NM1 C-terminus. In G1 this phosphorylation event stabilizes NM1 and prevents NM1 polyubiquitination by the E3 ligase UBR5 and proteasome-mediated degradation. We conclude that GSK3β-mediated phosphorylation of NM1 is required for pol I transcription activation.

## Author Summary

Nuclear actin and myosin are essential regulators of gene expression. At the exit of mitosis, nuclear myosin 1c (NM1) mediates RNA polymerase I (pol I) transcription activation and cell cycle progression by modulating assembly of the chromatin remodeling complex WICH with the subunits WSTF and SNF2h and, crucially, facilitating H3K9 acetylation by the histone acetyl transferase PCAF. The molecular mechanism by which NM1 is regulated remains however unknown. Here, we conducted a genome-wide screen and demonstrate that GSK3β is selectively coupled to the rDNA transcription unit. In embryonic fibroblasts lacking GSK3β there is a significant drop in rRNA synthesis levels and the rDNA is devoid of actin, NM1 and SNF2h. Concomitantly with a transcriptional block we reveal decreased levels of histone H3 acetylation by the histone acetyl transferase PCAF. At G1, transcriptional repression in the GSK3β knockout mouse embryonic fibroblasts, leads to NM1 ubiquitination by the E3 ligase UBR5 and proteasome-mediated degradation. We conclude that GSK3β suppresses NM1 degradation through the ubiquitin-proteasome system, facilitates NM1 association with the rDNA chromatin and transcription activation at G1. We therefore propose a novel and fundamental role for GSK3β as essential regulator of rRNA synthesis and cell cycle progression.

## Introduction

rRNA genes are transcribed by RNA polymerase I (pol I) into a large precursor (pre)-rRNA which is cleaved into 18S, 5.8S and 28S rRNAs for incorporation into ribosomal subunits [1], [2]. Pol I, in complex with the transcription initiation factor TIF1A, is first recruited to the gene promoter via the upstream binding factor (UBF) and the selectivity factor 1 (SL1) [3]. After promoter assembly, pol I transcription requires the synergy between actin and nuclear myosin 1c (NM1) [4], [5]. The interaction between pol I-associated actin with the chromatin-bound NM1 is required for transcription activation [6]–[9]. NM1 interacts with the chromatin through its C-terminal tail and it is also part of the multiprotein assembly B-WICH that contains the WICH chromatin remodeling complex with the subunits WSTF and the ATPase SNF2h but does not comprise actin [9]–[12]. While WSTF bookmarks the position of the chromatin remodeling complex on the rDNA transcription unit, NM1 interacts with SNF2h, stabilizes the WICH complex but, crucially, facilitates recruitment of the histone acetyl transferase (HAT) PCAF [9]. An important structural role has therefore been ascribed to NM1 that connects pol I with the chromatin through direct interactions with chromatin and the pol I-associated actin, respectively. This mechanism depends on the myosin ATPase activity. Further, this mechanism activates transcription by providing the permissive chromatin that in turn facilitates polymerase function across the active gene through modulating WICH assembly and PCAF recruitment [9]. At the exit of mitosis, this mechanism is critical for cell cycle progression when pol I transcription must be re-activated [9]. However, how NM1 is regulated at the onset of pol I transcription activation is not known.

GSK3β is a proline-directed serine/threonine kinase regulated by phosphorylation. The unphosphorylated form of GSK3β is enzymatically active [13], [14]. GSK3β is inactivated through activation of several signaling pathways including Wnt signaling that either leads to serine phosphorylation [15]–[17], or disrupts multiprotein complexes that contain GSK3β and its substrates [18]. GSK3β regulates cellular metabolism, the cytoskeleton and gene expression [16]. GSK3β also mediates cell cycle progression by phosphorylating pro-proliferative factors for degradation or by phosphorylating and stabilizing anti-proliferative factors. c-Myc is an example of short-lived proteins that is ubiquitinated in a GSK3β -dependent manner by the F-box protein Fbw7 and subsequently degraded by the proteasome [19]. GSK3β also controls expression of cyclin D1, which is phosphorylated to promote nuclear export and subsequent degradation [20]. In contrast, GSK3β-mediated phosphorylation of a single serine residue (Ser-118) in the estrogen receptor α leads to the stabilization of the receptor and protects it from proteasome-mediated degradation [21]. This dual mode of activity ensures that cell cycle progression, growth and proliferation are kept under tight regulation.

Previous work has shown a link between GSK3β and pol I-specific transcription factors [22]. Induction of granulocytic differentiation in murine myeloid cells results in the degradation of UBF via GSK3β and the ubiquitin/proteasome system [23]. Furthermore, enzymatically active GSK3β interacts with the member of the SL1 complex TAF_I_110 and suppresses pol I transcription in a H-RAS dependent manner [24]. GSK3β has therefore been suggested to suppress pol I transcription by repressing assembly of transcription-competent polymerase at rRNA gene promoter in transformed cells.

Here, we studied whether GSK3β has a more fundamental role in pol I transcription in non-transformed cells. A genome-wide screen showed that GSK3β is selectively distributed across the entire rDNA transcription unit. Further, GSK3β is required for rDNA association of numerous factors required for pol I transcription. In GSK3β^−/−^ mouse embryonic fibroblasts (MEFs) we found decreased levels of occupancy of actin, NM1 and SNF2h, at both promoter and transcribed sequences. These mechanisms, along with ultrastructural analysis of nucleoli in the GSK3β^−/−^ MEFs, correlate with decreased pol I transcription through loss of permissive chromatin. Further, in G1-arrested GSK3β^−/−^ MEFs, NM1 becomes specifically ubiquitinated by the E3 ligase UBR5 and degraded by the proteasome. These observations collectively suggest that GSK3β suppresses NM1 degradation through the ubiquitin-proteasome system, facilitates NM1 association with the rDNA chromatin and promotes pol I transcription activation at G1. We therefore propose a novel and fundamental role for GSK3β as a key regulator of rRNA synthesis.

## Results

### GSK3β associates with the rDNA chromatin

**Figure 1:**
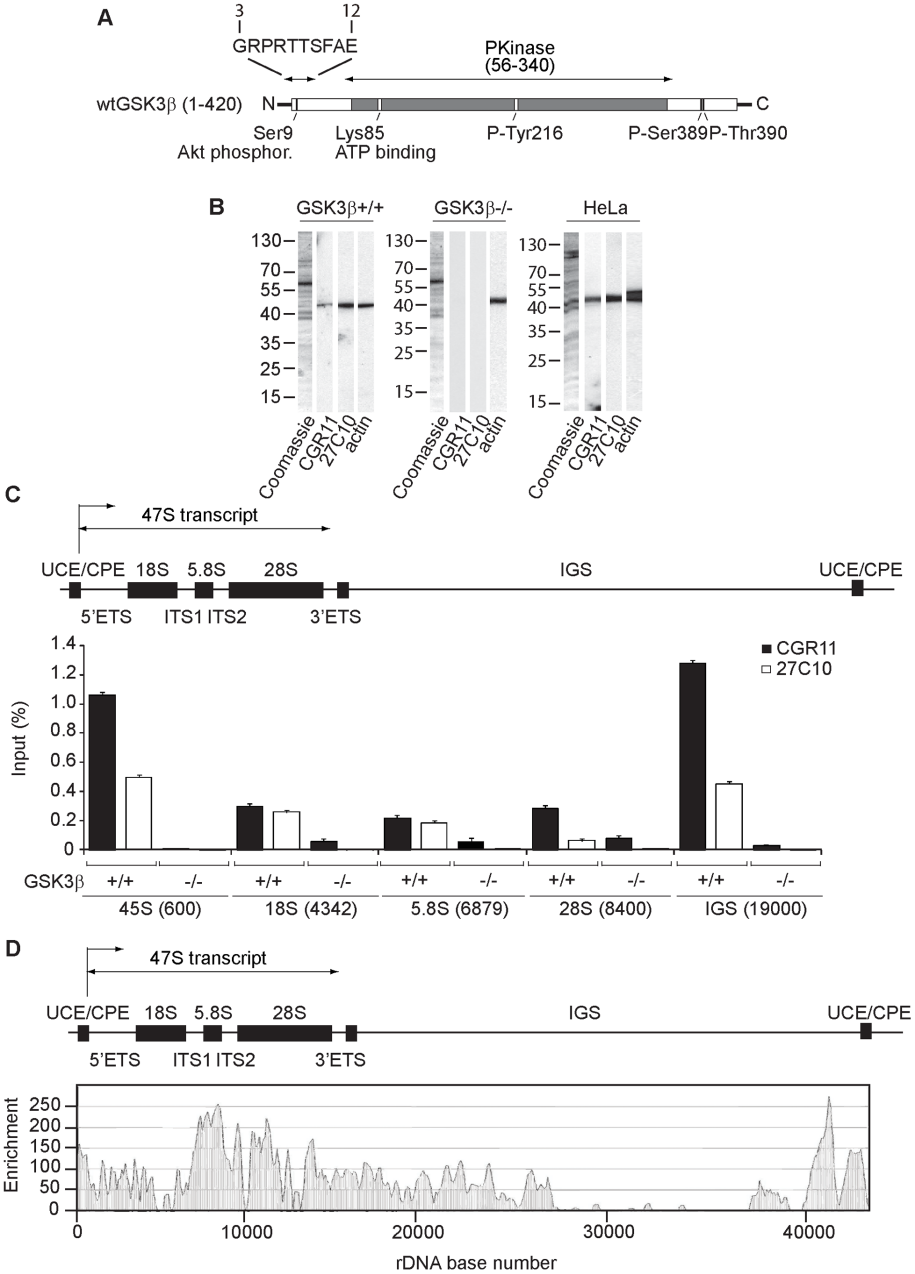
GSK3β distributes through the entire rDNA transcription unit, occupying the rRNA gene promoter and transcribed sequences. (**A**) Schematic representation of the primary structure of human GSK3β, including the N-terminal stretch of amino acids used as epitope for the GSK3β antibody CGR11. (**B**) Immunoblots of total lysates obtained from GSK3β^+/+^ MEFs, GSK3β^−/−^ MEFs and HeLa cells analyzed with the anti-GSK3β antibodies CGR11 and 27C10 and with an anti-actin antibody. (**C**) ChIP and qPCR on growing GSK3β^+/+^ MEFs and GSK3β^−/−^ MEFs at the rRNA gene promoter, 18S, 5.8S, 28S rDNA and IGS with the anti- GSK3β antibodies CGR11 and 27C10. Positions of all primers are indicated in bracket. The structure of individual mouse ribosomal rDNA repeat is shown to show the location of the different rDNA fragments analyzed. (**D**) ChIP-Seq performed on GSK3β^+/+^ MEFs. The previously sequenced mouse rDNA repeat BK000964 was utilized in our analysis procedure. The frequency of hits by sequences matching the region spanning the rDNA repeat sequence and IGS is shown by the resulting graph.

We confirmed the localization of GSK3β on the rDNA with a novel antibody termed CGR11 against the first nine N-terminal amino acids of the protein (Figure 1A). Since the epitope contains Serine 9, which is kept unphosphorylated in the active form of GSK3β [25], the CGR11 antibody is designed to preferentially target active GSK3β. The CGR11 antibody specifically detected a single protein of 48 kDa on immunoblots of nuclear extracts from HeLa cells and wild type mouse embryonic fibroblasts (GSK3β^+/+^ MEFs), similarly to the commercial pan- GSK3β antibody 27C10 (Figure 1B), but not in the GSK3β^−/−^ MEFs.

We used the GSK3β antibodies CGR11 and 27C10 to study GSK3β occupancy along the rDNA transcription unit by chromatin immunoprecipitation (ChIP). We prepared crosslinked chromatin from both GSK3β^+/+^ MEFs and GSK3β^−/−^ MEFs and subjected the chromatin to immunoprecipitations with the CGR11 and 27C10 antibodies. The precipitated DNA was analyzed by quantitative real-time PCR (qPCR) using primers amplifying fragments of 45S (promoter), 18S, 5.8S, 28S rDNA and the IGSs (intergenic sequences). The qPCR analysis shows that both antibodies precipitated rDNA from the chromatin isolated from GSK3β^+/+^ MEFs (Figure 1C). In contrast we did not get any signal when chromatin isolated from GSK3β^−/−^ MEFs was used in the immunoprecipitations (Figure 1C). We conclude that GSK3β specifically associates with the rDNA.

For further assessment of GSK3β protein occupancy throughout the rDNA we performed a genome-wide screen by ChIP followed by next-generation sequencing (ChIP-Seq). We subjected crosslinked chromatin isolated from GSK3β^+/+^ MEFs to immunoprecipitations with the CGR11 antibody and the DNA fragments were sequenced directly. Compared to background genomic binding levels the rDNA repeat showed GSK3β binding that was approximately two orders of magnitude higher. High levels of binding were found within the rRNA gene sequence, its upstream promoter elements and in the IGS extending 12 kb downstream of the rDNA (Figure 1D; Figure S1). In contrast large segments of the IGS appeared devoid of GSK3β binding (Figure 1D). The pattern of GSK3β binding across the rDNA transcription unit is similar to the binding patterns of pol I and UBF to the rDNA repeats [26]–[28]. Interestingly, very low levels of GSK3β binding to other genomic loci were detected (Table S1). Although the presence of GSK3β in the IGSs is not yet understood, the distribution of GSK3β all along the rDNA transcription unit, including externally transcribed sequences (ETSs), 18S, 5.8S, 28S and internally transcribed sequences (ITSs), suggests that GSK3β has a primary role in pol I transcription regulation.

### rDNA transcription activation is modulated by GSK3β

**Figure 2:**
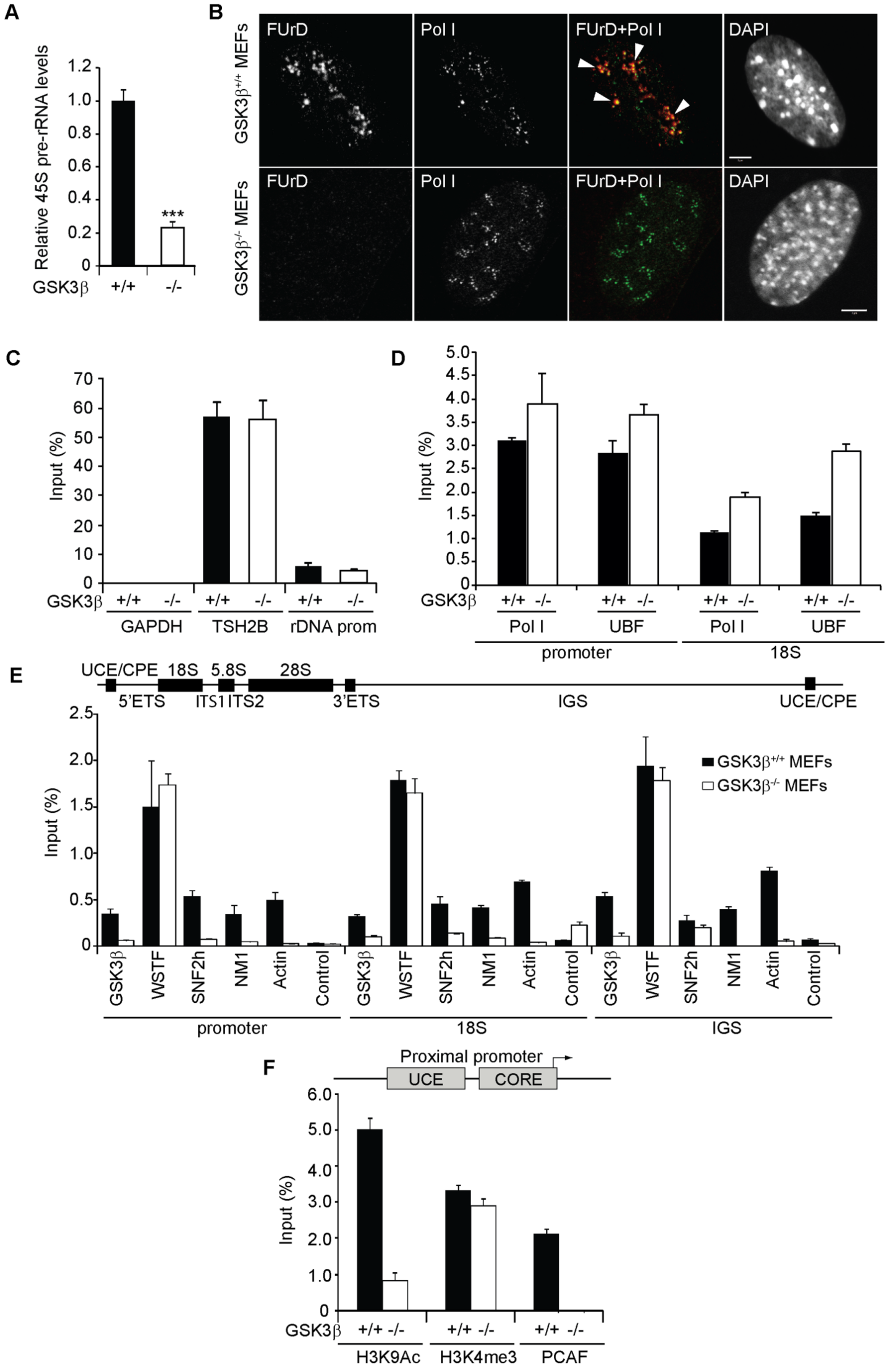
GSK3β regulates pol I transcription activation. (**A**) rRNA synthesis in GSK3β^+/+^ MEFs and GSK3β^−/−^ MEFs. For the analysis, relative 45S pre-rRNA levels were monitored from total RNA preparations by RT–qPCR using actin mRNA as internal control. Error bars represent the standard deviation of three independent experiments [p = 3.39e-05 (***)]. (**B**) FUrD incorporation assays on living GSK3β^−/−^ and GSK3β^+/+^ MEFs subjected to DRB treatment. Transcription was monitored by a short FUrd pulse to monitor incorporation into nascent nucleolar transcripts. After fixation, cells were co-stained with a fluorochrome conjugated anti-BrdU antibody to detect the incorporated FUrd and with a human auto-immune serum against pol I (S57299). Detection was by confocal microscopy. Scale bar, 5 ìm. (**C**) MeDIP and qPCR analysis on growing GSK3β^+/+^ MEFs and GSK3β^−/−^ MEFs performed with an antibody for 5-methylcytidine. qPCR analysis on the precipitated DNA was performed with primers amplifying rRNA gene promoter and reference genes TSH2B and GAPDH. (**D**) ChIP and qPCR on growing GSK3β^+/+^ MEFs and GSK3β^−/−^ MEFs at the rRNA gene promoter and 18S with the pol I specific autoimmune serum S57299 and an anti-UBF antibody. (**E**) ChIP and qPCR on growing GSK3β^+/+^ MEFs and GSK3β^−/−^ MEFs at the rRNA gene promoter, 18S and IGS with the anti-GSK3β antibody CGR11 and antibodies against WSTF, SNF2h, NM1, actin and non-specific rabbit IgGs. (**F**) ChIP and qPCR on growing GSK3β^+/+^ MEFs and GSK3β^−/−^ MEFs at the rRNA gene promoter with antibodies against H3K9Ac, H3K4me3 and PCAF.

To evaluate the possible involvement of GSK3β in rDNA transcription, we isolated total RNA from wild type and GSK3β^−/−^ MEFs and measured relative pre-rRNA levels by quantitative reverse transcription real time PCR (qRT-PCR). Using primers amplifying 45S pre-rRNA, in the GSK3β^−/−^ MEFs we detected a fivefold drop in the amount of nascent transcript relative to β-actin mRNA levels (Figure 2A). Twofold decrease in the levels of 45S pre-rRNA levels were also detected in HeLa cells subjected to GSK3β gene silencing by RNAi (Figure S2). To confirm these results in living cells, GSK3β^+/+^ MEFs and GSK3β^−/−^ MEFs were treated with DRB to selectively block RNA polymerase II transcription and then subjected to in situ run on assays. In these experiments incorporation of the cell permeable fluorine-conjugated UTP analogue FUrD was allowed for 10 minutes and the FUrD incorporated in nascent rRNA transcripts was monitored by immunofluorescence and confocal microscopy [29]. Consistent with the qRT-PCR analysis these experiments showed that in the absence of GSK3β, FUrD incorporation in nascent nucleolar transcripts was down-regulated (Figure 2B).

**Figure 3:**
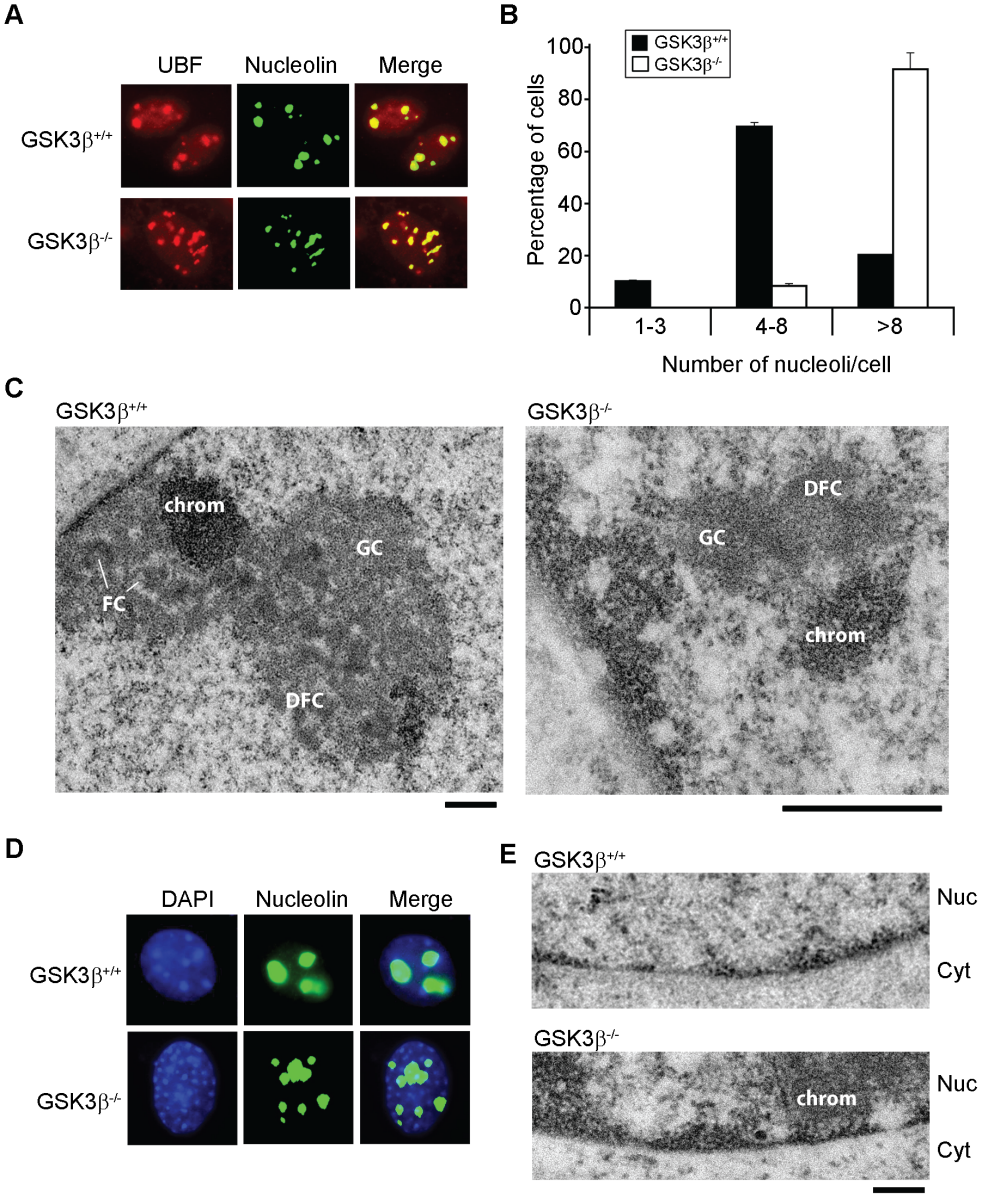
The effects of GSK3β knockout on the morphology of the nucleolus. (**A**) The size and number of nucleoli in GSK3β^+/+^ and GSK3β^−/−^ MEFs were analyzed in double-stained preparations using anti-UBF (red) and anti-nucleolin (green) antibodies as nucleolar markers. (**B**) Quantitative evaluation of the number of nucleoli per cell. The histogram shows the average number of nucleoli per cell based on the analysis of 212 GSK3β^+/+^ MEFs and 211 GSK3β^−/−^ MEFs, from two independent experiments. (**C**) The ultrastructure of the nucleolus in GSK3β^+/+^ and GSK3β^−/−^ MEFs analyzed by transmission electron microcopy. GSK3β^+/+^ nucleolus. DFC: dense fibrillar component; FC: fibrillar center; GC: granular component; chrom: dense chromatin. The magnification bars represent 0.5 µm. (**D**) GSK3β^+/+^ and GSK3β^−/−^ MEFs stained with an antibody against nucleolin (green) and counterstained with DAPI (blue) to visualize patterns of chromatin condensation. Dense chromatin of GSK3β^−/−^ MEFs is found in small patches that are often located at the nuclear periphery, whereas in GSK3β^+/+^ cells they are often larger and more centrally located. (**E**) Transmission electron microscopy images showing the accumulation of dense chromatin near the nuclear envelope in GSK3β^−/−^ MEFs. Nuc: nucleus; Cyt: cytoplasm. The bar represents 200 nm.

We next analyzed how GSK3β affects pol I transcription. We started by applying methylated DNA immunoprecipitation (MeDIP), for unbiased detection of methylated DNA [30], [31]. Genomic DNA obtained from GSK3β^+/+^ MEFs and GSK3β^−/−^ MEFs was randomly sheared by sonication and immunoprecipitated with a monoclonal antibody that recognizes 5-methylcytidine. qPCR analysis on the immunoprecipitated DNA using primers amplifying rRNA gene promoter show that the methylation levels did not change in the absence of GSK3β when compared to the reference TSH2B gene, a region of the histone H2B gene which is known to be methylated (Figure 2C). To determine whether the absence of GSK3β affected rDNA occupancy of the pol I machinery, we performed ChIP on crosslinked chromatin from GSK3β^+/+^ MEFs and GSK3β^−/−^ MEFs with a human autoimmune serum against active pol I (S57299), an antibody to UBF, and antibodies to actin, WSTF, SNF2h, NM1, GSK3β (CGR11) and non-specific rabbit IgGs. In GSK3β^−/−^ MEFs we detected modest increments in the amounts of promoter and 18S co-precipitated with the pol I and UBF antibodies (Figure 2D). In contrast, we detected drops in the amounts of promoter, 18S and IGSs co-precipitated with antibodies to actin, NM1 and SNF2h (Figure 2E). The WSTF antibody precipitated promoter, 18S and IGSs with similar efficiencies from both GSK3β^+/+^ MEFs and GSK3β^−/−^ MEFs chromatin (Figure 2E). These results indicate that as consequence of GSK3β knockout the rDNA occupancies of pol I machinery, actin and certain components of the B-WICH complex are altered. These changes were accompanied by decreased PCAF occupancy and H3K9 acetylation levels (Figure 2F). However, the levels of H3K4me3 were not altered. We conclude that in the GSK3β^−/−^ MEFs reduced levels of rRNA synthesis are primarily due to a chromatin state which is not compatible with transcription. Morphological analyses of nucleoli in GSK3β^−/−^ MEFs were consistent with this hypothesis. GSK3β^−/−^ and GSK3β^+/+^ MEFs were synchronized in G1 to avoid differences in cell cycle progression, double-stained with antibodies against nucleolin and UBF, and analyzed by fluorescence microscopy. The overall nuclear size and shape were similar in the two cell lines, but the GSK3β^−/−^ MEFs showed a significant increase in the number of nucleoli per cell, and the nucleoli were smaller (Figures 3A–B). The GSK3β^−/−^ nucleoli were UBF-positive, in accordance with our ChIP results (Figure 2D).

We also analyzed the ultrastructure of the nucleoli in GSK3β^−/−^ and GSK3β^+/+^ MEFs by transmission electron microscopy. In normal GSK3β^+/+^ MEFs, the nucleoli were well developed and showed a normal morphology with three typical components: dense fibrillar centers (DFCs), fibrillar component (FC) and granular component (GC). The GSK3β^−/−^ cells were instead characterized by reduced nucleoli that often lacked a well-defined compartmentalization and displayed reduced amounts of GC (Figure 3C). We could occasionally observe GSK3β^−/−^ MEFs with large nucleoli, but these nucleoli exhibited a highly vacuolated ultrastructure that was never observed in GSK3β^+/+^ nucleoli (Figure S3). Interestingly, the nucleolar alterations that we observed in GSK3β^−/−^ MEFs do not resemble the nucleolar disruption phenomenon that has been described in response to a variety of stress conditions [32]. The morphology of the GSK3β^−/−^ nucleoli and the fact that they contain UBF, support instead the idea that a larger number of nucleolar organizer regions (NORs) becomes activated in GSK3β^−/−^ MEFs than in GSK3β^+/+^ MEFs. However, these numerous GSK3β^−/−^ NORs do not engage in efficient rRNA production and fail to assemble fully structured nucleoli. The morphological analysis of GSK3β^−/−^ and GSK3β^+/+^ MEFs also revealed differences in the patterns of chromatin condensation between the two cell types. Staining of GSK3β^−/−^ MEFs with DAPI revealed the existence of small patches of dense chromatin scattered throughout the nucleoplasm, often in association with the nuclear periphery, whereas GSK3β^+/+^ MEFs were characterized by fewer and larger areas of densely packaged chromatin (Figure 3D). This difference was confirmed at the ultrastructural level (Figure 3E).

In summary, the morphological analyses reveal severe defects in nucleolar function and chromatin organization and together with the molecular analyses presented above support the conclusion that GSK3β contributes to pol I transcription activation by indirectly inducing a permissive chromatin state.

### GSK3β phosphorylates and stabilizes NM1 at early G1

**Figure 4:**
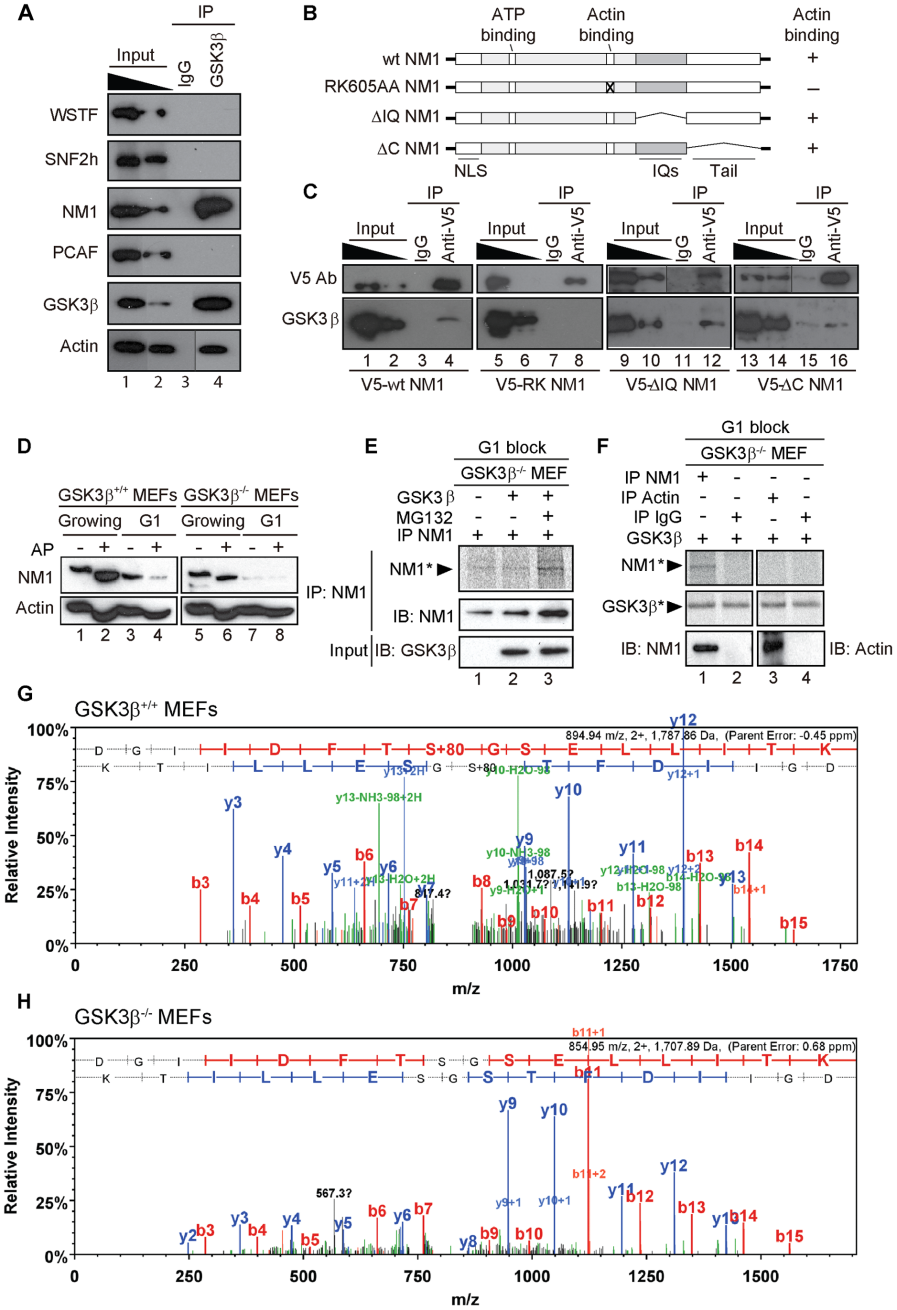
GSK3β phosphorylates NM1. (**A**) GSK3β, NM1 and actin are co-precipitated from nuclear protein extracts prepared from growing GSK3β^+/+^ MEFs. Bound proteins were detected on immunoblots with antibodies against WSTF, SNF2h, NM1, PCAF, GSK3β (CGR11) and actin. 10% of the input is shown in Lane 1. IP, immunoprecipitation. (**B**) Schematic representation of V5-tagged wt and mutated NM1 constructs stably expressed in HEK293T cell lines. (**C**) Co-precipitations of GSK3β from total lysates obtained from HEK293T cells stably expressing wt and mutated V5-tagged NM1 constructs as indicated. 10% of the input is shown. IP, immunoprecipitation. (**D**) Lysates were prepared from growing GSK3β^+/+^ MEFs and GSK3β^−/−^ MEFs or from GSK3β^+/+^ MEFs and GSK3β^−/−^ MEFs arrested in G1 by serum starvation. Where indicated extracts were subjected to alkaline phosphatase (AP) treatment. Lysates were analyzed on immunoblots for NM1 and actin. (**E**) Kinase assays were performed on lysates from G1-arrested GSK3β^−/−^ MEFs untreated or treated with the proteasome inhibitor MG132, supplemented with γ-^33^P-ATP. Where indicated the lysates were incubated with recombinant GSK3β. To monitor NM1 phosphorylation, the lysates were subjected to immunoprecipitations with anti-NM1 antibodies. Phosphorylated NM1 was detected by phosphorimaging against the levels of unphosphorylated NM1 detected on immunoblots. (**F**) Kinase assays were performed on endogenous NM1 or actin immunoprecipitated from lysates of G1-arrested GSK3β^−/−^ MEFs treated with MG132; after immunoprecipitations the beads were washed and incubated with γ-^33^P-ATP and recombinant GSK3β. Phosphorylated NM1 (NM1*) and autophosphorylated GSK3β (GSK3β*) were detected by phosphorimaging. The immunoprecipitated endogenous NM1 and actin were detected on immunoblots. Non-specific IgGs were used as negative control for the immunoprecipitations. (**G–H**) Tandem MS spectra of phosphoprylated and non-phosphorylated peptide DGIIDFTSGSELLITK identified within the primary NM1 sequence immunoprecipitated from G1-arrested lysates of GSK3β^+/+^ MEFs and GSK3β^−/−^ MEFs, respectively.

Actin, NM1, SNF2h and PCAF levels along the rDNA transcription unit are dependent on GSK3β (Figure 2E–F). To test whether GSK3β interacts with actin, NM1, SNF2h, WSTF or PCAF, we applied immunoprecipitations to nuclear lysates from GSK3β^+/+^ MEFs. Briefly, we incubated nuclear lysates with the anti- GSK3β antibody CGR11. Analysis of the immunoprecipitated fractions by immunoblotting showed that endogenous GSK3β co-precipitated NM1 as well as actin whereas SNF2h, PCAF, and WSTF were not co-immunoprecipitated (Figure 4A). These results show that in the nucleus GSK3β is part of the same complex with NM1 and actin.

To evaluate whether GSK3β targets any specific regions of NM1, we used HEK293T cell lines stably expressing V5-tagged wild-type NM1 (V5-wtNM1), a V5-tagged NM1 mutant with impaired actin binding function (V5-RK605AA NM1) as well as V5-tagged deletion constructs that lack IQ motifs (V5-ΔIQ NM1) or the tail domain (V5-ΔC NM1) [8], [9] (Figure 4B). We subjected total lysates from each of the above cell lines to immunoprecipitations with an anti-V5 antibody to pull down the NM1 constructs. Analysis of the co-immunoprecipitated proteins on immunoblots with the CGR11 antibody showed specific co-precipitations of endogenous GSK3β with V5-wtNM1 but not with the V5-RK605AA NM1 (Figure 4C). Further, GSK3β co-precipitated with V5-ΔIQ NM1 and with lower efficiency, also with V5-ΔC NM1 (Figure 4C). Interestingly, a recent study supports the possibility of a direct interaction with NM1 as three putative GSK consensus sites were identified in the C-terminus of the human NM1 [33]. Our present results suggest that NM1 and GSK3β are part of the same complex and further indicate that the association is negatively affected when NM1 cannot interact with actin.

To start evaluating whether GSK3β phosphorylates NM1, we resolved extracts from growing GSK3β^+/+^ MEFs or GSK3β^−/−^ MEFs by phosphate affinity SDS PAGE [34]. In these assays, extensive protein phosphorylation is revealed by comparing changes in the protein electrophoretic mobility on immunoblots of lysates treated or untreated with alkaline phosphatase. The results of immunoblots of growing GSK3β^+/+^ MEFs lysates for NM1 showed specific gel retardations which were lost upon phosphatase treatment (Figure 4D, lanes 1, 2). Similar results were obtained upon analysis of GSK3β^−/−^ MEFs lysates (Figure 4D, lanes 5, 6), altogether suggesting that in growing cells NM1 is extensively phosphorylated but not by GSK3β. We next applied phosphate affinity SDS PAGE to resolve lysates from GSK3β^+/+^ MEFs or GSK3β^−/−^ MEFs blocked in G1 by serum starvation (see also Figure S4). Even though we did not reveal the same extent of gel retardation as in growing cells, upon alkaline phosphatase treatment on wild type lysates we observed considerable reduction in the amount of NM1 (Figure 4D, lanes 3,4). Remarkably, analysis of immunoblots of lysates from the GSK3β^−/−^ MEFs blocked in G1 showed decreased NM1 levels independent of the alkaline phosphatase treatment (Figure 4D, lanes 7, 8). In support of this observation, analysis on immunoblots of lysates from GSK3β^−/−^ MEFs blocked in G1 by contact inhibition [35] independently revealed a similar drop in the NM1 protein levels (see also Figure S5A–B). Immunoblots performed on the phosphate affinity SDS PAGE for actin revealed that endogenous actin is phosphorylated, but phosphorylation appears to be independent of GSK3β since it was not affected in either growing or G1-blocked GSK3β^−/−^ MEFs (Figure 4D). Furthermore, in contrast to NM1, the expression levels of endogenous actin were not affected in growing or G1-arrested GSK3β^−/−^ MEFs. We therefore conclude that at G1 NM1 is specifically stabilized by GSK3β and this regulation possibly occurs through a direct interaction.

**Figure 5:**
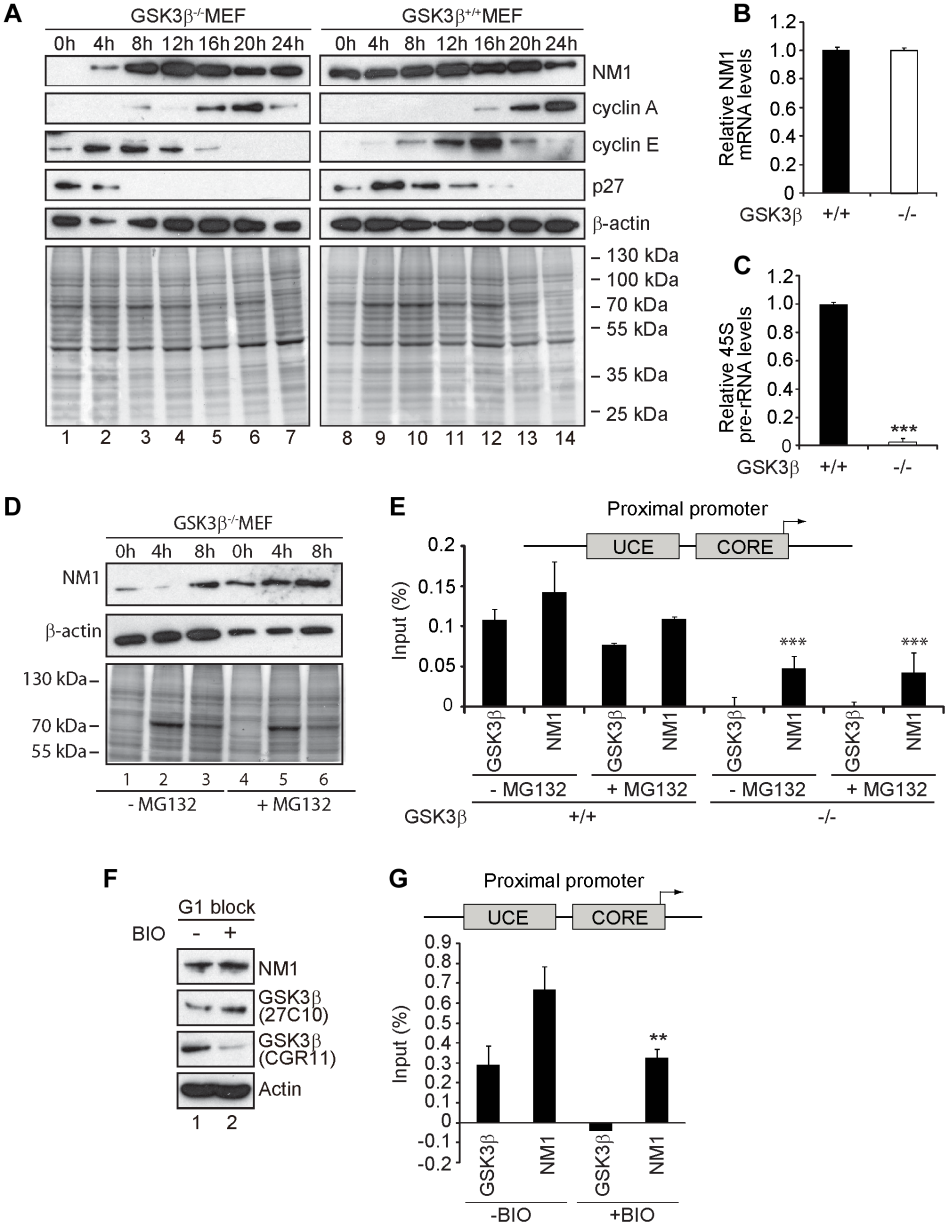
GSK3β-dependent NM1 phosphorylation suppresses proteasome mediated degradation and mediates association with chromatin. (**A**) Cell cycle profile analyzed at the indicated time points, after release from a G1 arrest by serum starvation, on immunoblots of the corresponding lysates for NM1, cyclin A, cyclin E, p27 and β-actin. (**B**) Relative NM1 mRNA levels in GSK3β^+/+^ MEFs and GSK3β^−/−^ MEFs monitored by RT-qPCR using β-tubulin mRNA as internal control. (**C**) rRNA synthesis in GSK3β^+/+^ MEFs and GSK3β^−/−^ MEFs arrested in G1 by serum starvation. For the analysis, relative 45S pre-rRNA levels were monitored from total RNA preparations by RT-qPCR using tubulin mRNA as internal control [p = 3.2e-09 (***)]. (**D**) Lysates from GSK3β^−/−^ MEFs untreated or treated with the proteasome inhibitor MG132, released from a G1 block were collected at the indicated time points and analyzed on immunoblots for NM1 and β-actin. (**E**) ChIP and qPCR analysis on chromatin isolated from GSK3β^+/+^ MEFs and GSK3β^−/−^ MEFs synchronized in G1, untreated or treated with MG132, at the rRNA gene promoter with antibodies against NM1 and GSK3β (CGR11). Significances p(−MG132) = 2.2e-05 (***) and p(+MG132) = 3.0e-05 (***) were respectively calculated against the NM1 values obtained in GSK3β^+/+^ MEFs not treated with MG132. (**F**) Immunoblots of total lysates from GSK3β^+/+^ MEFs untreated or treated with the kinase inhibitor BIO. Analysis was performed with antibodies to NM1, actin, and the GSK3β antibodies 27C10 and CGR11 as indicated. (**G**) ChIP and qPCR analysis on chromatin isolated from GSK3β^+/+^ MEFs at G1, untreated or treated with BIO, at the rRNA gene promoter with antibodies against NM1 and GSK3β (CGR11). The significance p = 0.009 (**) was calculated against the NM1 values obtained in GSK3β^+/+^ MEFs not treated with BIO.

We next investigated whether GSK3β directly phosphorylates NM1. To start addressing this point, we prepared G1 lysates from GSK3β^−/−^ MEFs, untreated or treated with the proteasome inhibitor MG132, under which conditions the NM1 levels are rescued (see also Figure 5D). Lysates were supplemented with γ-^33^P-ATP and with purified recombinant GSK3β. Following incubation, endogenous NM1 was immunoprecipitated, resolved by SDS PAGE and visualized by phosphorimaging (Figure 4E). The results show that a fraction of the immunoprecipitated NM1 was phosphorylated by recombinant GSK3β in the presence of MG132 (Figure 4E). To confirm that endogenous NM1 is a substrate for GSK3β, we subjected lysates from G1-arrested GSK3β ^−/−^ MEFs treated with MG132 to immunoprecipitations with anti-NM1 or anti-actin antibodies. After the immunoprecipitations, the beads were washed and the bound NM1 or actin were incubated with γ-^33^P-ATP and with purified recombinant GSK3β. Following incubation, the endogenous NM1 and actin were eluted from the beads and resolved by SDS PAGE. Analysis by phosphorimaging shows that a radioactive band was detected only for NM1 but not for actin (Figure 4F), indicating that NM1 is a direct substrate for GSK3β. Consistent with previous observations [36], in the same assay, a degree of GSK3β autophosphorylation was also detected. To further endorse the specificity of these results and identify potential phosphorylation sites in the NM1 primary sequence, we immunoprecipitated endogenous NM1 from nuclear extracts of MG132-treated GSK3β^+/+^ MEFs and GSK3β^−/−^ MEFs arrested in G1. The immunoprecipitated protein fraction was resolved by SDS-PAGE and subjected to in gel digestion. The resulting peptides were extracted and analyzed by tandem mass spectrometry. Analysis of the immunoprecipitated NM1 from the G1 GSK3β^+/+^ MEFs nuclear lysate identified the peptide DGIIDFTSGSELLITK in both its phosphorylated (Figure 4G) and non-phosphorylated state (Figure S6) with mascot ion scores of 61 and 94, respectively. The MS/MS data indicates that within the above peptide the phosphorylation is present at Serine 8 which in the mouse full length NM1 amino acid sequence corresponds to the Serine 1020 located in the NM1 C-terminal tail (Accession number, Q9WTI7-3). In contrast, the same analysis performed on the endogenous NM1 immunoprecipitated from the G1 GSK3β^−/−^ MEFs nuclear lysate identified the non-phosphorylated peptide DGIIDFTSGSELLITK with a mascot ion score of 78 (Figure 4H) but did not reveal its phosphorylated form. These results show that Ser-1020 is directly phosphorylated by GSK3β in early G1.

We conclude that the endogenous NM1 is a bona fide phosphorylation substrate for GSK3β. Phosphorylation specifically targets the NM1 C-terminus at Ser-1020 and occurs in G1. We suggest that at the exit of mitosis GSK3β phosphorylation stabilizes NM1 from proteasome-mediated degradation.

### GSK3β-mediated phosphorylation facilitates NM1 association with rDNA at G1

Cell cycle profiling by flow cytometry and NM1 steady state expression analysis in GSK3β^+/+^ MEFs and GSK3β^−/−^ MEFs after release from a G1 block confirmed a specific down regulation of NM1 (Figure 5A; Figure S7). In the absence of GSK3β NM1 down-regulation is at the protein level since qRT-PCR analysis of the relative NM1 mRNA levels in the GSK3β^−/−^ MEFs were not altered in comparison to those in the GSK3β^+/+^ MEFs (Figure 5B). Furthermore, we isolated total RNA from wild type and GSK3β^−/−^ MEFs blocked in early G1 and measured relative 45S pre-rRNA levels by qRT-PCR. Using both serum starvation and contact inhibition to arrest cells in G1, we detected significant drops in the amount of nascent transcript relative to β-tubulin mRNA levels (Figure 5C; Figure 5SC).

We next synchronized GSK3β^−/−^ MEFs in G1 by serum starvation and treated with the proteasome inhibitor MG132. After release from the G1 block, total lysates were collected at 0 h, 4 h and 8 h and analyzed for NM1 protein levels on immunoblots. In lysates from untreated GSK3β^−/−^ MEFs, NM1 expression was down at 0 h and 4 h after release from the G1 block and was rescued around 8 h after the release (Figure 5D). In contrast, immunoblots of lysates from MG132-treated GSK3β^−/−^ MEFs showed a marginal increase in the NM1 protein levels between 0 h and 8 h after the G1 block release (Figure 5D), altogether suggesting selective degradation of NM1 by the proteasome at early G1. Since treatment with MG132 rescued the levels of NM1, we further evaluated whether by inhibiting the proteasome we could also rescue the ability of NM1 to bind to the rDNA chromatin. We therefore performed ChIP on crosslinked chromatin from GSK3β^+/+^ and GSK3β^−/−^ MEFs blocked in G1, treated or untreated with MG132, using antibodies to NM1 and GSK3β (CGR11). The precipitated DNA was analyzed by qPCR with primers specific for the rDNA promoter. In the GSK3β^−/−^ MEFs the NM1 antibodies precipitated the promoter 2-fold less efficiently (Figure 5E), possibly due to NM1 degradation by the proteasome. NM1 occupancy on the promoter was not restored even in the presence of MG132 (Figure 5E). On the contrary, in ChIP experiments performed on chromatin from GSK3β^+/+^ MEFs at G1 the anti-NM1 antibody co-precipitated the promoter, independently of the MG132 treatment (Figure 5E). These results show that at G1, rescuing the NM1 protein levels by proteasome inhibition does not restore the ability of NM1 to efficiently bind the chromatin in the absence of GSK3β. To find out whether GSK3β-mediated phosphorylation of NM1 modulates association with the rDNA, we treated GSK3β^+/+^ MEFs arrested at G1 with the cell-permeable GSK3β inhibitor 6-bromoindirubin-30-oxime (BIO) [37], [38]. On immunoblots of lysates of BIO-treated GSK3β^+/+^ MEFs the reactivity of CGR11 to GSK3β was considerably decreased whereas the pan-GSK3β antibody 27C10 and the antibodies to actin and NM1 were not affected by the BIO treatment (Figure 5F). Using the CGR11 antibody we performed ChIP on crosslinked chromatin from BIO-treated GSK3β^+/+^ MEFs to monitor occupancies of NM1 and active GSK3β at the rDNA promoter. The qPCR results show that upon inhibition of the GSK3β kinase activity with BIO, GSK3β does not co-precipitate rDNA and under the same conditions, NM1 shows a 50% drop in rDNA binding (Figure 5G).

We conclude that at G1 GSK3β phosphorylates NM1 to facilitate NM1 association with the rDNA chromatin while simultaneously protecting NM1 from degradation by the proteasome.

### At G1 GSK3β phosphorylates NM1 to suppress ubiquitination by the E3 ligase UBR5

**Figure 6:**
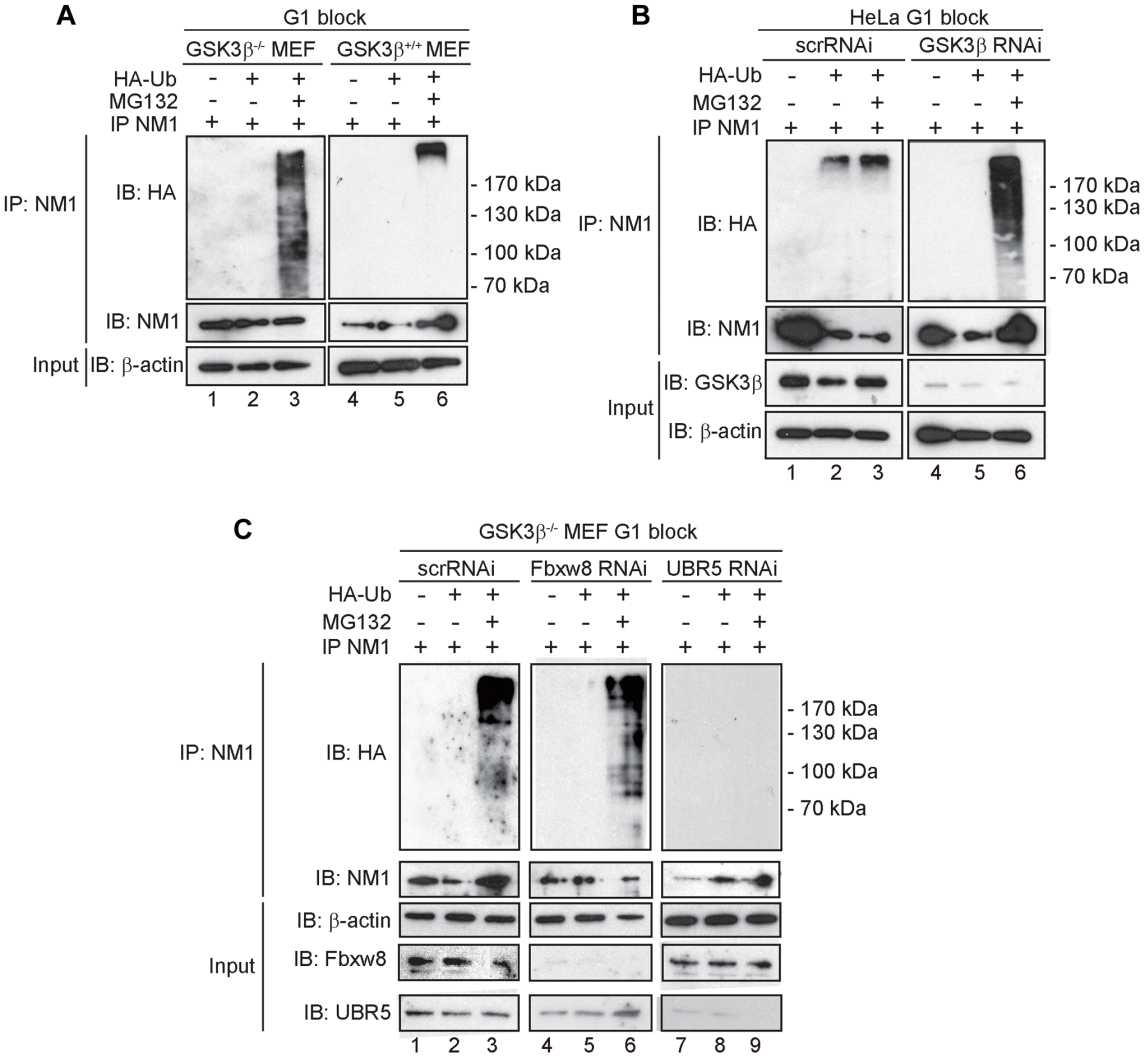
At G1, NM1 is ubiquitinated in a GSK3β-dependent manner by the E3 ligase UBR5. (**A**) Lysates prepared from GSK3β^+/+^ MEFs and GSK3β^−/−^ MEFs at G1 transiently expressing HA-tagged ubiquitin, treated with MG132 where indicated, were subjected to immunoprecipitations with the anti-NM1 antibody and the co-immunoprecipitated fractions were analyzed on immunoblots for HA-tagged ubiquitin. (**B**) Lysates from HeLa cells synchronized in G1 co-transfected with GSK3β RNAi oligonucleotides or scrambled scrRNAi oligonucleotides and transiently expressing HA-tagged ubiquitin. Where indicated lysates were obtained from HeLa cells treated with MG132. Immunoprecipitations were performed from all lysates with the anti-NM1 antibody and the co-immunoprecipitated fractions were analyzed on immunoblots for HA-tagged ubiquitin. (**C**) Lysates from G1-blocked GSK3β^−/−^ MEFs were subjected to RNAi-mediated gene silencing of the E3 ligases UBR5 and Fbxw8 or to scrRNAi oligonucleotides transiently expressing HA-tagged ubiquitin. The lysates were subjected to immunoprecipitations with the anti-NM1 antibody and the co-immunoprecipitated fractions were analyzed on immunoblots for HA-tagged ubiquitin.

We next determined whether NM1 is a GSK3β-dependent substrate for ubiquitination. For this purpose cells were transiently transfected with a plasmid encompassing a HA-tagged version of the ubiquitin open reading frame. Following expression of the HA-ubiquitin, cells were arrested in G1 by serum starvation and treated with MG132. We prepared total lysates and subjected them to immunoprecipitations with the anti-NM1 antibody. The fractions of co-immunoprecipitated proteins were analyzed on immunoblots for HA-tagged ubiquitin. The results show that in contrast to GSK3β^+/+^ MEFs, in G1 lysates from GSK3β^−/−^ MEFs treated with MG132, NM1 becomes polyubiquitinated (Figure 6A). NM1 polyubiquitination was not detected in lysates from growing GSK3β^−/−^ MEFs even in the presence of MG132 (Figure S8A), whereas in lysates prepared from growing GSK3β^+/+^ MEFs or HEK293T cells NM1 appears to be polyubiquitinated (Figure S8B–C). To confirm these results in an independent cellular system, HeLa cells were incubated with a master mix containing the HA-tagged ubiquitin plasmid and siRNA oligonucleotides for specific GSK3β gene silencing (GSK3β RNAi) or control scrambled siRNA oligonucleotides (scrRNAi) (Figure 6B; Figure S2). HeLa cells were maintained at G1 by serum starvation and were treated with MG132. Lysates were subjected to immunoprecipitations with anti-NM1 antibodies and the co-immunoprecipitated protein fractions were analyzed on immunoblots for HA-tagged ubiquitin. The results show a marked increase in the levels of endogenous NM1 polyubiquitination in lysates of GSK3β-silenced HeLa cells treated with MG132 (Figure 6B, lanes 3 and 6). We conclude that NM1 polyubiquitination is dependent on GSK3β only at G1.

To identify possible E3 ligases involved in NM1 ubiquitination, we subjected nuclear lysates from untreated or MG132-treated GSK3β^+/+^ MEFs to immunoprecipitations with antibodies to NM1. The co-immunoprecipitated proteins were subjected to mass spectrometry analysis by nLC-MS/MS. Within the subset of co-immunoprecipitated proteins we found two candidate E3 ligases, UBR5 and F-box/WD repeat-containing protein 8 (Fbxw8) (Table S2). Remarkably, UBR5 and Fbxw8 were not co-precipitated with NM1 from MG132-treated GSK3β^+/+^ MEFs nuclear lysates (Table S3), suggesting a functional association with NM1. To find out whether UBR5 and Fbxw8 target NM1 for ubiquitination in a GSK3β-dependent manner at G1, we silenced the UBR5 and Fbxw8 genes in the GSK3β^−/−^ MEFs expressing HA-tagged ubiquitin and maintained at G1. After treatment with MG132, lysates were subjected to immunoprecipitations with anti-NM1 antibodies and the co-immunoprecipitated protein fractions were analyzed on immunoblots for HA-tagged ubiquitin. The results show that silencing Fbxw8 did not affect the level of NM1 polyubiquitination compared to control scrRNAi (Figure 6C). On the contrary NM1 polyubiquitination was not observed upon UBR5 gene silencing (Figure 6C). We conclude that in the absence of GSK3β NM1 polyubiquitination is specifically mediated by UBR5 at G1.

In summary, NM1 phosphorylation by GSK3β blocks NM1 ubiquitination by UBR5 and degradation by the proteasome, leads to NM1 association with the chromatin and promotes rDNA transcription activation at G1.

## Discussion

By interacting with some components of the SL1 complex, in cells transformed with oncogenic H-RAS GSK3β functions as negative regulator of rDNA transcription suppressing assembly of transcription-competent pol I at the gene promoter [24]. Here we show for the first time that GSK3β also has a positive role on the basal mechanism that leads to activation of rRNA synthesis. Genomic analysis of GSK3β by ChIP-Seq showed that GSK3β selectively distributes across the entire rDNA transcription unit and to a certain degree, GSK3β also binds to intergenic sequences. This association with the gene is shown to be functional since in the GSK3β knockout MEFs we found a fivefold reduction in rRNA synthesis levels, which correlated with increased levels of pol I occupancy at the rRNA gene promoter. Furthermore, results from the ChIP analysis showed decreased occupancy for actin, NM1 and SNF2h at the promoter and across the gene. We have recently shown that NM1 binds the rDNA chromatin, to promote the activation of pol I transcription by stabilizing the B-WICH complex, such that it can subsequently recruit the HAT PCAF. These mechanisms contribute to the permissive chromatin required for pol I transcription activation [9]. These results are compatible with the pol I transcriptional drop observed in the GSK3β knockout MEFs where the rRNA gene promoter is almost quantitatively devoid of the HAT PCAF and displays a fourfold down-regulation in the levels of H3K9 acetylation. Local impairment of H3 acetylation is accompanied by moderate impairment of the chromatin remodeling function due to the absence of SNF2h at the gene promoter. The morphological analysis of GSK3β^−/−^ MEFs suggests that in the absence of GSK3β activity the cell activates additional NORs, probably in an attempt to compensate for the reduced rRNA production. The GSK3β^−/−^ nucleoli are not fully functional but do recruit nucleolin and UBF, which indicates that these nucleoli are functional to some extent, in agreement with our molecular analysis. The GSK3β^−/−^ MEFs also display differences in the overall patterns of chromatin condensation. These chromatin changes could either be a direct consequence of impaired GSK3β function or an indirect response to the reduced ribosome biosynthetic capacity of the cell. In any case, the severe structural alterations observed in the nuclei of GSK3β^−/−^ cells support the important role of GSK3β in rRNA biogenesis. We conclude that GSK3β contributes to transcription activation and maintenance by regulating local rDNA chromatin modifications.

Our results suggest that across the rDNA transcription unit GSK3β performs its regulatory function by targeting pol I-associated factors. GSK3β is a promiscuous enzyme [39] that phosphorylates serine or threonine at position 4 of the consensus sites S/TXXXS/T[PO3] [40], [41], but also single Ser residues outside the above mentioned consensus sequence [21]. About 20% of the mammalian proteome contains multiple putative GSK3 phosphorylation sites [33]. UBF has, for instance, five putative GSK consensus sites and it is directly phosphorylated by GSK3β in vitro and in vivo [23]. Both NM1 and actin were also found to be potential GSK3β substrates [33]. Accordingly, we found that GSK3β co-precipitated NM1 and actin from nuclear lysates, but not SNF2h, WSTF or PCAF. This suggests that GSK3β is part of the same complex with actin and NM1. We found however that only NM1 is directly phosphorylated by GSK3β and that NM1 phosphorylation by GSK3β occurs on a single Serine residue (Ser-1020), located within the murine NM1 C-terminal tail domain. Mass spectrometry analysis identified a single NM1 phosphopeptide in G1-arrested GSK3β^+/+^ MEFs but not in the GSK3β^−/−^ MEFs. We cannot exclude the presence of other GSK3β phosphorylation sites; Ser-1020 phosphorylation however, seems to occur outside the canonical GSK3β consensus site. Considering that the state of NM1 phosphorylation is not dependent on GSK3β in growing cells, we conclude that the Ser-1020 phosphorylation by GSK3β exclusively occurs in G1.

**Figure 7:**
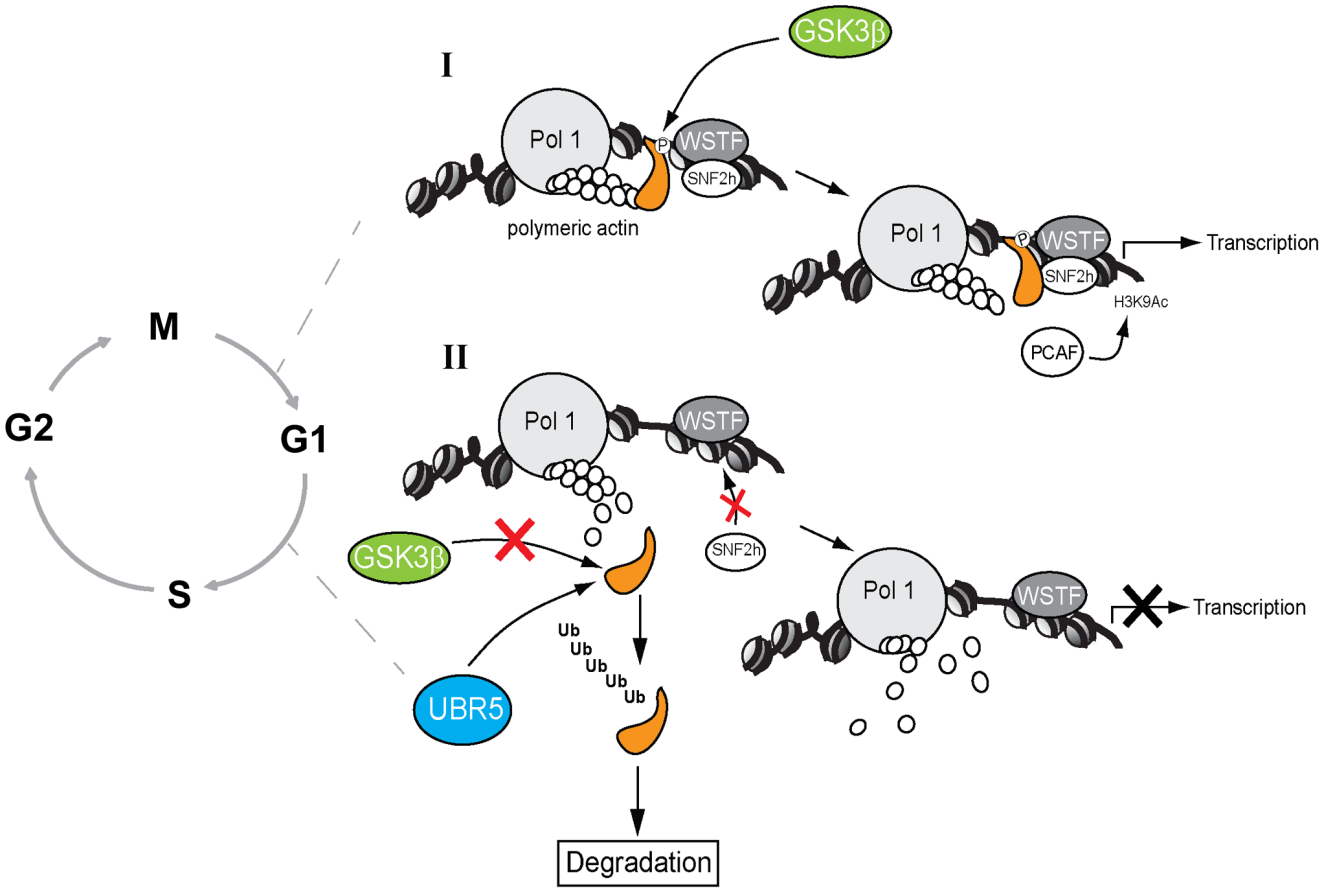
A speculative model in which GSK3β phosphorylates the NM1 C-terminal tail at G1. (**I**) In the presence of GSK3β, NM1 is phosphorylated and binds to rDNA chromatin. This phosphorylation event triggers a domino effect that leads to stabilization of the actomyosin complex and B-WICH multi-protein assembly on the rDNA. This mechanism leads to recruitment of PCAF, maintains the levels of H3K9 acetylation and activates transcription. (**II**) When GSK3β does not phosphorylate NM1, NM1 becomes polyubiquitinated by UBR5 and degraded by the proteasome. Consequently, the WICH complex is not assembled on the chromatin. At G1 NM1 degradation leads to suppression of pol I transcription and alterations in cell cycle progression.

GSK3β-dependent phosphorylation can lead to either stabilization of the substrates or further polyubiquitination and degradation by the proteasome [42], [43]. UBF phosphorylation by GSK3β promotes UBF degradation by the ubiquitin-proteasome system, concomitantly with differentiation of myeloid cells [23]. Our results therefore indicate that at early G1, GSK3β phosphorylates NM1 to prevent NM1 polyubiquitination and degradation by the proteasome. Consistently, in the absence of GSK3β, we discovered that at G1 NM1 is polyubiquitinated by UBR5 and rapidly degraded. UBR5, also termed EDD/hHyd, is an E3 ligase that targets the N-terminus of its substrates and interacts with GSK3β [44], [45]. UBR5 resides in the nucleolus and it is known to associate with SIRT7 [46]. Furthermore, UBR5 seems to have a huge impact on cell cycle progression. UBR5 regulates S-phase and G2/M DNA damage checkpoints, it induces cell cycle arrest by increasing p53 levels and has been recently implicated in cellular proliferation [47]–[49]. Since in G1-arrested GSK3β^−/−^ MEFs we discovered an almost quantitative drop in the levels of nascent rRNA, we now hypothesize that GSK3β-mediated phosphorylation protects NM1 from UBR5-mediated polyubiquitination, and this mechanism is important for pol I transcription activation and progression through the cell cycle (Figure 7).

A key question is why NM1 phosphorylation by GSK3β is important for rRNA synthesis at a specific temporal window of the cell cycle. We recently demonstrated that NM1 binds the rDNA chromatin through its C-terminal tail, interacts with the pol I-associated actin and this interaction is dependent on the myosin ATPase cycle. Association with the chromatin stabilizes actin-binding and allows for the establishment of permissive chromatin required for pol I transcription activation and cell cycle progression [9]. In the GSK3β knockout cells arrested in G1, the rRNA gene promoter displays reduced NM1 levels even when the NM1 protein expression is rescued by MG132 treatment. Similarly, NM1 promoter occupancy levels drop after inhibition of GSK3β activity in living cells by treatment with BIO. The NM1 C-terminal tail is necessary for chromatin association [9]. It is therefore possible that phosphorylation by GSK3β is a primary requirement for NM1 to bind the rRNA gene promoter. Since NM1 association with the rDNA is a condition for actin occupancy [9], it is tempting to speculate that by enhancing association of NM1 with the chromatin, phosphorylation at Ser-1020 indirectly stabilizes the actomyosin complex on the rDNA; this may be achieved by tethering of GSK3β to NM1 through actin.

How the NM1 phosphorylation by GSK3β is restricted to the G1 phase of the cell cycle is not known and deserves further investigation. Our working model is however that at G1 GSK3β-mediated NM1 phosphorylation unleashes a domino effect that maintains the actin-NM1 complex and B-WICH assembly on the rDNA chromatin. This mechanism therefore stabilizes the multiprotein complex that contains GSK3β and its substrate NM1 and contributes to defining the structure and organization of the pol I machinery with respect to its chromatin template for pol I transcription activation and cell cycle progression.

In summary we propose a novel gatekeeping function for GSK3β across the rDNA, where GSK3β targets NM1 and consequently controls local chromatin modifications compatible with rRNA synthesis by instructing G1 cells to slow down NM1 degradation. In the GSK3β knockouts, cell cycle progression and in particular, the transition to S-phase occurs more rapidly than in wild type cells. The dual mode of GSK3β activity ensures that cell cycle progression is kept under tight regulation in proliferating cells [20]. It is therefore tempting to speculate that NM1 is a novel proliferative factor that is directly stabilized by GSK3β at G1 and it is likely to be altered concomitantly with inactivation of GSK3β, possibly in response to intracellular signaling.

## Methods

### Antibodies

The CGR11 antibody against GSK3β was designed as peptide specific polyclonal antibody against the N-terminal amino acid sequence GRPRTTSFAE and affinity purified against the same epitope (Agrisera AB, Sweden). The anti-GSK3β antibody 27C10 was purchased from Cell Signaling (9315). Antibodies against WSTF (ab50850), SNF2h (ab3749), H3K9Ac (ab10812), Ki67 (ab15580) and the non-specific rabbit IgGs (ab46540) were from Abcam. The mouse anti-PCAF (sc13124) and anti-UBF antibodies, the rabbit anti-UBF (sc9131), anti-Nucleolin (sc-13057) and anti-Cyclin D1 (sc-718) antibodies were purchased from Santa Cruz Biotech, whereas the anti-β-actin antibody (clone AC74) was from Sigma Aldrich. The V5 epitope antibody (A190-120A) was purchased from Bethyl Laboratories. The human autoimmune sera S57299 specific for the RPA194 pol I subunit was a kind gift of U. Scheer (Wurzburg University, Germany) [10] and the antibody against NM1 has previously been characterized [6]. The monoclonal antibody to bromouridine triphosphate (BrdU) to monitor FUrd incorporation was from Sigma Aldrich. Species-specific secondary antibodies conjugated to Cy2, Alexa 488, Alexa 568, Alexa 594 or Texas-Red were purchased from Invitrogen and Jackson ImmunoResearch. The secondary antibodies conjugated to FITC and Texas-Red were from DakoCytomation. DNA was revealed by DAPI staining (300 nM for 3 min at room temperature, RT).

### Cell culture, reagents and flow cytometry

The GSK3β ^+/+^ MEFs and GSK3β^−/−^ MEFs were a kind gift from J.R. Woodgett (University of Toronto, Canada). MEFs, HeLa and HEK293T cells were grown in DMEM medium (Gibco), supplemented with 10% foetal bovine serum (Gibco) and a 1% penicillin/streptomycin cocktail (Gibco). For synchronization in G1, subconfluent cells were grown in serum-free media for 24 hours or grown until contact inhibition [35]. The cells were released from the G1 block by adding back serum. Where indicated, GSK3β^+/+^ MEFs, GSK3β^−/−^ MEFs and HeLa cells were incubated with MG132 (Cayman Chemical, ref no 10012628) to a final concentration of 40 µM for 3 h at 37°C. GSK3β^+/+^ MEFs were also incubated with BIO (Sigma Aldrich, B1686) to a final concentration of 1 µM for 24 hrs at 37°C. HEK293T cells and HEK293T cells constitutively expressing V5-tagged wtNM1 and RK605AA NM1 point mutant, as well as ΔIQ NM1 and ΔC NM1 deletion mutants were previously characterized [9] and are gifts of I. Grummt (University of Heidelberg, Germany). The plasmid expressing the HA-tagged Ubiquitin was a gift of O. Sangfelt (Karolinska Institutet, Sweden).

Flow cytometry (FACS) was performed as described [9]. Briefly, cells were collected by trypsinization and fixed in 70% ethanol on ice for 15 min. The DNA was stained with propidium iodine (PI) solution containing 50 µg/ml PI, 0.1 mg/ml RNasaA and 0.05% Trition X-100 in PBS (phosphate buffer saline) at 37°C for 40 min. Cells were then FACS on FACSCalibur (Becton Dickinson) and 10000 cells were counted. The experiment was repeated three times.

### ChIP assays and qPCR analysis

ChIP on growing or synchronized GSK3β^+/+^ MEFs, GSK3β^−/−^ MEFs was performed as previously described [9]. Briefly, formaldehyde cross-linked chromatin was obtained from growing cells and from early G1 cells, treated or untreated with BIO (1 µM) and MG132 (40 µM) as indicated. Cross-linked chromatin was immunoprecipitated with antibodies to pol I (S57299), UBF, WSTF, SNF2h, NM1, Actin, H3K9Ac, PCAF, GSK3β (CGR11 and 27C10) and non-specific rabbit IgGs. DNA-protein complexes were analyzed by qPCR with specific primers amplifying multiple regions of the rRNA gene, including promoter, 18S, 5.8S, 28S and IGS (see Table S4 for sequences). qPCR was performed using SYBR-green from Applied Biosystems according to the manufacturer's instructions. The primer concentration was 2.5 mM and the samples analyzed by Rotor-Gene 6000 series software 1.7. The PCR conditions were: hold 95°C for 3 minutes, followed by cycles of 95°C for 3 seconds, 60°C for 20 seconds, 72°C for 3 seconds. The results were analyzed using an average of Ct of IgG as background. The 2ΔCt of each sample in triplicates was related to the 2ΔCt of the input sample.

### ChIP-Seq, sequencing data alignment and analysis

For ChIP-Seq analysis, crosslinked chromatin from GSK3β^+/+^ MEFs was subjected to immunoprecipitations with the GSK3β antibody CGR11. 5 ng of precipitated DNA was used to prepare sequencing libraries at the Bejing Genome Institute (Hong Kong) using the Illumina HiSeq 2000 platform. For sequencing data alignment and analysis, the current assembly of mouse reference genome is missing the ribosomal rDNA repeats, of which there are approximately 400, located on chromosomes 12, 14 and 15. To compensate for this we utilized the procedure of Zentner et al (2011) [28] and constructed a custom reference sequence that contained a non-masked rDNA repeat, taken from BK000964, which was added to the end of chromosome 12 of the MM9 assembly. This allows for the identification and mapping of sequences present within the rDNA repeat region that are associated with the ChIP pulldown. Without the addition of the rDNA to the assembly these sequences would be removed from the analysis pipeline. The method has previously been shown to be a robust technique to identify regions within the genomic rDNA region that are associated with transcription factors, silencing or enhancing factors or histone modifications. The ChIP-Seq data sets are available in the Gene Expression Omnibus (GEO) database with accession number GSE57153. The analysis procedure involved the use of the SOAP2 program to map the reads to the constructed reference genome. Sequences with more than two mismatches were discarded from further analysis. The resulting individual sequences were remapped back to the annotated UCSC MM9 reference sequence, which allows for the identification of peaks corresponding to the levels of association of the ChIP target with those loci.

### Methylated DNA immunoprecipitation assays (MeDIP)

MeDIP assays were carried out essentially as previously described [30], [31]. In brief, genomic DNA was extracted using the Qiagen QIAmp DNA kit from GSK3β^+/+^ and GSK3β^−/−^ MEFs. DNA was sonicated to make 200–500 bp fragments and subsequently denatured. Immunoprecipitation with anti-5-methyl-cytodin (Abcam) antibody was done overnight. The complexes were captured with protein A Sepharose. The methylated DNA was finally eluted with minElute PCR purification kit (Qiagen) and qPCR for the rDNA promoter region was run. Bars represent percent of input.

### Analysis of protein-protein interactions

Immunoprecipitation assays were performed as previously described [9]. Endogenous GSK3β from nuclear extracts of growing GSK3β^+/+^ MEFs nuclear lysates were incubated with the CGR11 antibody or control non-specific rabbit IgGs. Constitutively expressed V5-tagged wtNM1, RK605AA NM1, ΔIQ NM1 and ΔC NM1 mutants from HEK293T cells lysates were incubated with the anti-V5 epitope antibody and control non-specific rabbit IgGs. The antibodies were subsequently precipitated with Protein G Sepharose (Invitrogen). The beads were washed with 1XPBS supplemented with 1 mM PMSF, 0.2 % NP-40 and then resuspended in SDS-loading buffer and heat denatured. Bound proteins were resolved by SDS-PAGE and analyzed on immunoblots for GSK3β, NM1, WSTF, SNF2h, PCAF, actin or V5. Endogenous NM1 from nuclear extracts of growing GSK3β^+/+^ MEFs treated or untreated with MG132 (40 µM for 3 hrs at 37°C) were incubated with the CGR11 antibody or control non-specific rabbit IgGs. The co-immunoprecipitated protein fractions were resolved by SDS-containing gel electrophoresis and in gel digested with trypsin (minus the heavy and light chain gel sections). The tryptic peptides were analyzed on a RSLC nanoLC system coupled to a Velos I system (LTQ Orbitrap Velos Pro).

### Kinase assays and analysis of endogenous phosphorylated proteins

To monitor phosphorylation of the endogenous NM1 protein, GSK3β^−/−^ MEFs were arrested in G1 by serum starvation and treated with 40 µM MG132 for 3 hrs at 37°C. Lysates were prepared in 20 mM Hepes pH 7.4, 0.05 mM ATP, 10 mM MgCl2, 1 mM dithiothreitol, 2 mM sodium orthovanadate, and then incubated with 5 µCi of γ-^33^P-ATP and recombinant GSK3β (Abcam) for 30 min at 30°C. The lysates were next subjected to immunoprecipitations with the anti-NM1 antibody as previously described [9]. The immunoprecipitated NM1 was resolved by SDS PAGE and the proportion of phosphorylated NM1 within the immunoprecipitated protein fraction was detected by phosphorimaging. Alternatively, the kinase assays were performed on immunoprecipitated NM1 or actin from lysates of GSK3β^−/−^ MEFs treated with MG132 still coupled to the Sepharose beads. Briefly, the beads were washed with 1X PBS containing 0.5% NP-40 and supplemented with γ-^33^P-ATP and recombinant GSK3β for 30 min at 30°C. The immunoprecipitated protein fraction was detected by phosphorimaging and immunoblotting for NM1 and actin.

Phosphate affinity gel electrophoresis for detection of endogenous NM1 and actin phosphorylation was performed as previously described [9]. Lysates prepared from growing or G1 synchronized GSK3β^+/+^ MEFs and GSK3β^−/−^ MEFs were separated by 8% SDS-PAGE containing 25 µM Phos-tagTM AAL-107 according to the manufacturer's instructions (MANAC Incorporated) and 50 µM MnCl_2_, and transferred to a PVDF membrane using a transfer buffer containing 25 mM Tris, 86 mM glycine and 10% methanol. Immunoblots were analyzed for NM1 and actin. Where indicated extracts were subjected to alkaline phosphatase treatment as described in the instruction manual provided by the manufacturer (New England Biolabs). For identification of phosphorylated residues, NM1 was immunoprecipitated from nuclear lysates prepared from GSK3β^+/+^ MEFs and GSK3β^−/−^ MEFs, resolved by SDS PAGE and in gel digested with trypsin. The tryptic peptides were analyzed by tandem mass spectrometry.

### Ubiquitin assays

GSK3β^+/+^ MEFs, GSK3β^−/−^ MEFs, HeLa and HEK293T cells on 10 cm dishes were transfected with 7–10 ng of a plasmid expressing HA-tagged ubiquitin using Lipofectamine 2000 as described in the instruction manual (Invitrogen). Following 24 h expression cells were treated with 40 µM MG132 for 3 hrs at 37°C and lysed in SDS containing lysis buffer (1 % SDS, 25 µl saturated NEM in PBS). Lysates were denatured and the SDS diluted to 0.1% with 1X PBS. Lysates were subjected to immunoprecipitations with the anti-NM1 antibody overnight at 4°C and precipitated with Protein G Sepharose. The beads were washed in 0.5% NP-40 buffer, resuspended in SDS-containing buffer and heat denatured. Samples were resolved by SDS-PAGE and transferred on PVDF membrane for immunodetection of ubiquitin with anti-HA epitope antibody. Where indicated, ubiquitination assays were performed on GSK3β^−/−^ MEFs and HeLa cells subjected to GSK3β, UBR5 or Fbxw8 gene silencing by RNAi (see below).

### GSK3β, UBR5 and Fbxw8 gene silencing by RNAi

For the GSK3β gene silencing, HeLa cells were subjected to GSK3β RNAi oligonucleotides (target sequence 5′ GGACCCAAAUGUCAAACUA) or control scrambled RNAi (scrRNAi) oligonucleotides (5′ UCGUUGCAGGAUAUGUAGUUUUU). GSK3β gene silencing duplexes and control scrambled versions were purchased from Dharmacon and applied by transfection with Lipofectamine RNAiMax (Invitrogen) at a final concentration of 400 pmol for 24 hrs. For the UBR5 and Fbxw8 genes silencing, GSK3β^−/−^ MEFs were subjected to RNAi oligonucleotides (Dharmacon) to UBR5 (target sequence 5′-GGGUGUACAUUCUUUAAUA) and Fbxw8 (target sequence 5′-CGCCAAGGAGCACACAUUA) applied by transfection with Lipofectamine RNAiMax at a final concentration of 400 pmol for 24 hrs.

### Transcription assays

To reveal active pol I transcription foci, living GSK3β^+/+^ MEFs and GSK3β^−/−^ MEFs grown on cover slips were pre-incubated with DMEM supplemented with 75 µM DRB (Sigma Aldrich) for 1 h. The FURD (Sigma-Aldrich) was then added to a final concentration of 2 mM and cellular uptake was allowed for up to 10 min [9], [29]. Cells were fixed with a 3.7% formaldehyde solution in PBS at room temperature and permeabilized with a 0.5% Triton X-100 solution in PBS. For detection of incorporated FURD, fixed cells were incubated with a mouse monoclonal antibody to BrdU followed by a Cy3-conjugated goat anti-mouse secondary antibody. Fluorescence images were obtained from a confocal microscope (Zeiss LSM meta) with 63X oil objective NA 1.3. Images were collected and analyzed using the LSM software.

For analysis of nascent pre-rRNA, total RNA was extracted from growing GSK3β^+/+^ MEFs, GSK3β^−/−^ MEFs and GSK3β-silenced HeLa cells with the TRI reagent as specified by the manufacturer (Sigma). 1 ng of RNA was reversed transcribed and analysis by qRT-PCR with specific primers amplifying mouse and human 45S pre-rRNA relative to β-actin mRNA. qRT-PCR was performed using SYBR-green from Applied Biosystems according to the manufacturer's instructions (see also above for further details). Where indicated the same analysis was performed on nascent 45S pre-rRNA in GSK3β^+/+^ MEFs, GSK3β^−/−^ MEFs synchronized in G1, both by serum starvation and contact inhibition. Relative 45S pre-rRNA levels were measured by qRT-PCR against the levels of β-tubulin mRNA (primers sequences are shown in Table S4). The qRT-PCR values are shown as bars diagrams. Error bars represent the standard deviation of three independent experiments. Significances were obtained by Student's T-test, two-sample, equal variance.

### Immunofluorescence

For immunolocalization of nucleolin and UBF, GSK3β^+/+^ MEFs and GSK3β^−/−^ MEFs cells were grown on coverslips to subconfluence and arrested in G1 by growing in serum-free medium for 24 hours. The cells were fixed with 4% formaldehyde in PBS for 15 min, permeabilized with 0.1% Triton X-100 in PBS for 13 min at room temperature and stained with primary antibodies against Nucleolin and UBF following standard procedures. Secondary antibodies conjugated to FITC and Texas-Red were used to visualize Nucleolin and UBF, respectively. The slides were mounted in Vectashield containing DAPI (Vector Laboratories), and examined and photographed with an Axioplan fluorescence microscope (Carl Zeiss). Cells in random areas of the preparations were classified into three groups according to the number of positively stained nucleoli per cell (one to three, four to eight, more than eight).

### Transmission electron microscopy

Subconfluent GSK3β^+/+^ MEFs and GSK3β^−/−^ MEFs cells were arrested in G1 by growing in serum-free medium for 24 hours. The cells were pelleted and fixed with 2% glutaraldehyde (Merck) in Sorensen's phosphate buffer, washed with Sorensen's buffer, and embedded in 2% low melting point agarose. The agarose blocks were cut into small pieces, dehydrated in a graded series of ethanol at room temperature, and embedded in Agar 100 resin (Agar Scientific Ltd). The embedded cell pellets were cut into 50 nm thin sections, mounted on 100 mesh copper grids, and stained with 2% uranyl acetate in 50% ethanol for 5 min at room temperature. The specimens were examined and photographed in a transmission electron microscope Tecnai G2 Spirit BioTwin (FEI Company) at 80 kV. Photoshop software (Adobe) was used for the preparation of composite images and for adjustment of intensity and contrast.

## Supporting Information

Figure S1 ChIP and qPCR on growing GSK3β^+/+^ MEFs and GSK3β^−/−^ MEFs at the rRNA gene promoter (45S-2) and proximal (IGS-1) and distal (IGS-2) positions across the IGS with the anti-GSK3β antibody CGR11 for further validation of the ChIP-Seq analysis (see Table S4). The values are presented as the percentage of the input signal for each primer pair. The structure of individual mouse ribosomal rDNA repeat is shown to show the location of the different rDNA fragments analyzed.
(TIF)

Figure S2 GSK3β gene silencing by RNAi in HeLa cells. (**A**) GSK3β steady state expression levels on immunoblots of lysates prepared from control (scrRNAi) and GSK3β-silenced HeLa cells. (**B**) Densitometric quantification of GSK3β steady state protein expression relative to β-actin. (**C**) rRNA synthesis in growing HeLa cells subjected to GSK3β gene silencing by RNAi. For the analysis, relative 45S pre-rRNA levels were monitored from total RNA preparations by RT–qPCR using β-actin mRNA as internal control. Error bars represent the standard deviation of three independent experiments.
(TIF)

Figure S3 The ultrastructure of the nucleus in the GSK3β knockout MEFs. (**A**) Overview images showing the morphology of the cell nuclei of GSK3β^+/+^ and GSK3β^−/−^ MEFs, as indicated. In GSK3β^+/+^ MEFs, the dense chromatin is typically concentrated in a few large domains (arrow), whereas the nucleus of GSK3β^−/−^ MEFs contains a larger number of smaller chromatin patches (arrows) that are often associated with nucleolar material. The magnification bar represents 1 µm. (**B**) An example of GSK3β^−/−^ MEFs displaying large and very vacuolated nucleoli. The magnification bar represents 200 nm.
(TIF)

Figure S4 GSK3β^+/+^ MEFs and GSK3β^−/−^ MEFs subjected to serum starvation. (**A**) Cell cycle profile for growing and time-course serum starvation (16 h, 20 h, 24 h, 48 h) performed on propidium iodide-stained GSK3β^+/+^ MEFs and GSK3β^−/−^ MEFs by FACS. (**B**) Immunoblots of lysates obtained from growing and serum starved GSK3β^+/+^ MEFs and GSK3β^−/−^ MEFs at the time points 16 h, 20 h, 24 h, 48 h, using antibodies to Ki67, cyclin D1, NM1, GSK3β and β-actin.
(TIF)

Figure S5 GSK3β^+/+^ MEFs and GSK3β^−/−^ MEFs blocked in G1 by contact inhibition. (**A**) Cell cycle profile for growing and G1-arrested GSK3β^+/+^ MEFs and GSK3β^−/−^ MEFs by FACS on propidium iodide-stained cells. (**B**) Immunoblots of lysates from GSK3β^+/+^ MEFs and GSK3β^−/−^ MEFs arrested in G1 by contact inhibition using antibodies for NM1, cyclin D1, and β-actin. (**C**) rRNA synthesis in GSK3β^+/+^ MEFs and GSK3β^−/−^ MEFs arrested in G1 by contact inhibition. For the analysis, relative 45S pre-rRNA levels were monitored from total RNA preparations by RT–qPCR using tubulin mRNA as internal control [p = 5.4e-05, ***].
(TIF)

Figure S6 Tandem MS spectrum of non-phosphorylated peptide DGIIDFTSGSELLITK identified within the primary NM1 sequence immunoprecipitated from G1-arrested nuclear lysate of GSK3β^+/+^ MEFs.
(TIF)

Figure S7 Cell cycle profile analyzed at the indicated time points after release from a G1 arrest by serum starvation using FACS on propidium iodide-stained GSK3β^+/+^ MEFs and GSK3β^−/−^ MEFs.
(TIF)

Figure S8 In growing cells NM1 is not ubiquitinated in a GSK3β-dependent manner. (**A**) Lysates were prepared from growing GSK3β^−/−^ MEFs transiently expressing HA-tagged ubiquitin. The lysates were subjected to immunoprecipitations with the anti-NM1 antibody and the co-immunoprecipitated fractions were analyzed on immunoblots for HA-tagged ubiquitin. Lane 1, immunoprecipitationf from growing GSK3β^−/−^ MEFs which do not express HA-tagged ubiquitin; lane 2, immunoprecipitations from untreated growing GSK3β^−/−^ MEFs expressing HA-tagged ubiquitin; lane 3, immunoprecipitations from growing GSK3β^−/−^ MEFs expressing HA-tagged ubiquitin treated with MG132. (**B**) Lysates were prepared from growing GSK3β^+/+^ MEFs transiently expressing HA-tagged ubiquitin. The lysates were subjected to immunoprecipitations with the anti-NM1 antibody and the co-immunoprecipitated fractions were analyzed on immunoblots for HA-tagged ubiquitin. Lane 1, immunoprecipitations from growing GSK3β^+/+^ MEFs which do not express HA-tagged ubiquitin; lane 2, immunoprecipitations from growing GSK3β^+/+^ MEFs expressing HA-tagged ubiquitin; lane 3, immunoprecipitations from growing GSK3β^+/+^ MEFs expressing HA-tagged ubiquitin treated with MG132. (**C**) Lysates were prepared from growing HEK293T cells transiently expressing HA-tagged ubiquitin. The lysates were subjected to immunoprecipitations with the anti-NM1 antibody and the co-immunoprecipitated fractions were analyzed on immunoblots for HA-tagged ubiquitin. Lane 1, immunoprecipitations from growing HEK293T cells which do not express HA-tagged ubiquitin; lane 2, immunoprecipitations from untreated growing HEK293T cells expressing HA-tagged ubiquitin; lane 3, immunoprecipitations from growing HEK293T cells expressing HA-tagged ubiquitin treated with MG132.
(TIF)

Table S1 GSK3β binding genome-wide. Mouse genomic regions corresponding to high levels of deep sequencing hits from the ChIP-Seq analysis.
(DOC)

Table S2 Mass spectrometry identification of the fraction of proteins co-immunoprecipitated with the anti-NM1 antibody from total lysates of untreated GSK3β^+/+^MEFs.
(XLS)

Table S3 Mass spectrometry identification of the fraction of proteins co-immunoprecipitated with the anti-NM1 antibody from total lysates of GSK3β^+/+^ MEFs treated with the proteasome inhibitor MG132.
(XLS)

Table S4 Sequences of mouse and human oligonucleotide primers used in the qPCR and qRT PCR analyses. For primer sequences, see references [7], [9], [50].
(DOC)
