# Supplementary figures and images for: Glycogen Synthase Kinase (GSK) 3β Phosphorylates and Protects Nuclear Myosin 1c from Proteasome-Mediated Degradation to Activate rDNA Transcription in Early G1 Cells
Source: PLoS Genet. 2014 Jun 5;10(6):e1004390. doi: 10.1371/journal.pgen.1004390 (PMC4046919; doi:10.1371/journal.pgen.1004390)

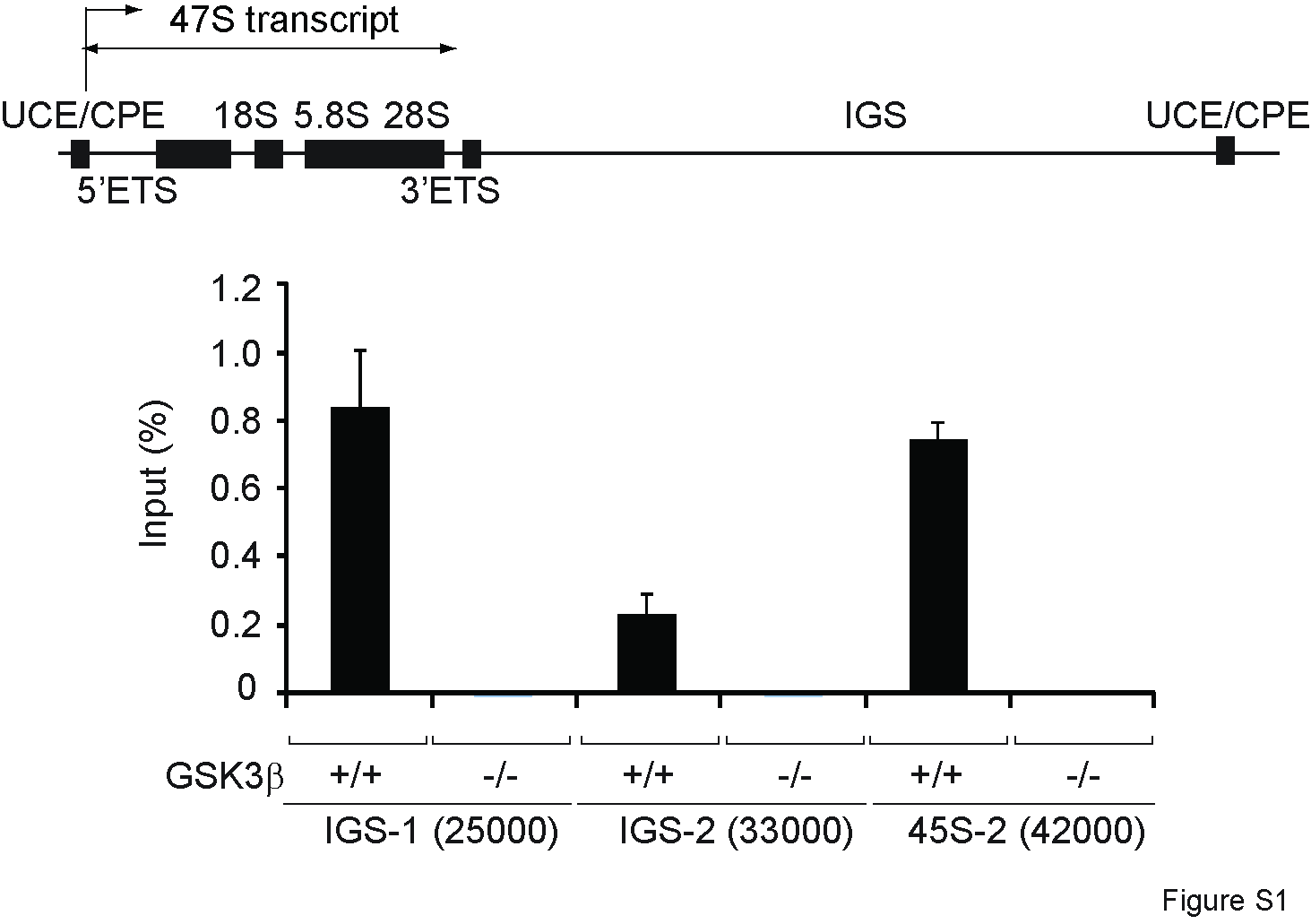

Supplement: Figure S1 — ChIP and qPCR on growing GSK3β+/+ MEFs and GSK3β−/− MEFs at the rRNA gene promoter (45S-2) and proximal (IGS-1) and distal (IGS-2) positions across the IGS with the anti-GSK3β antibody CGR11 for further validation of the ChIP-Seq analysis (see Table S4). The values are presented as the percentage of the input signal for each primer pair. The structure of individual mouse ribosomal rDNA repeat is shown to show the location of the different rDNA fragments analyzed. (TIF) [file pgen.1004390.s001.tif]

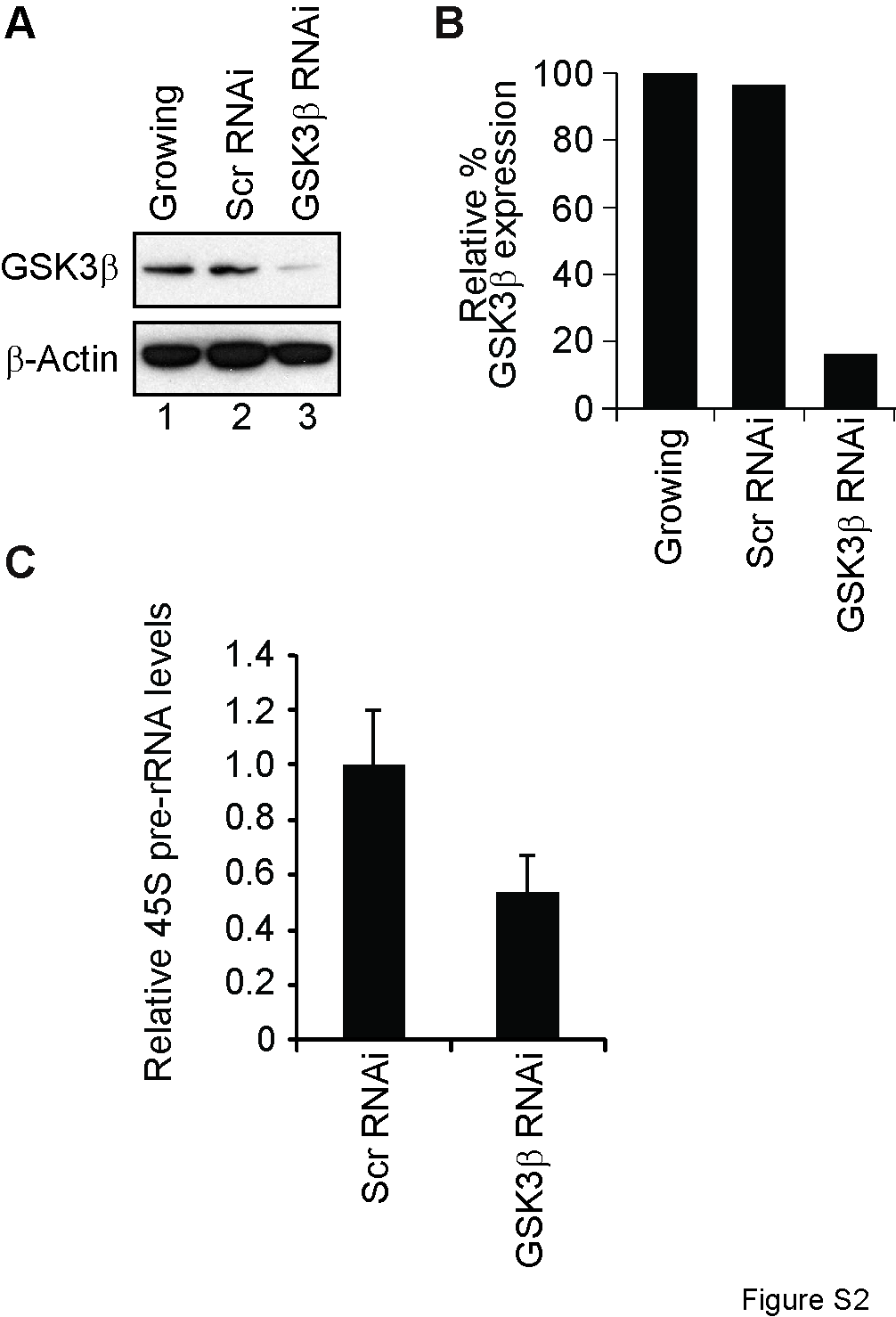

Supplement: Figure S2 — GSK3β gene silencing by RNAi in HeLa cells. (A) GSK3β steady state expression levels on immunoblots of lysates prepared from control (scrRNAi) and GSK3β-silenced HeLa cells. (B) Densitometric quantification of GSK3β steady state protein expression relative to β-actin. (C) rRNA synthesis in growing HeLa cells subjected to GSK3β gene silencing by RNAi. For the analysis, relative 45S pre-rRNA levels were monitored from total RNA preparations by RT–qPCR using β-actin mRNA as internal control. Error bars represent the standard deviation of three independent experiments. (TIF) [file pgen.1004390.s002.tif]

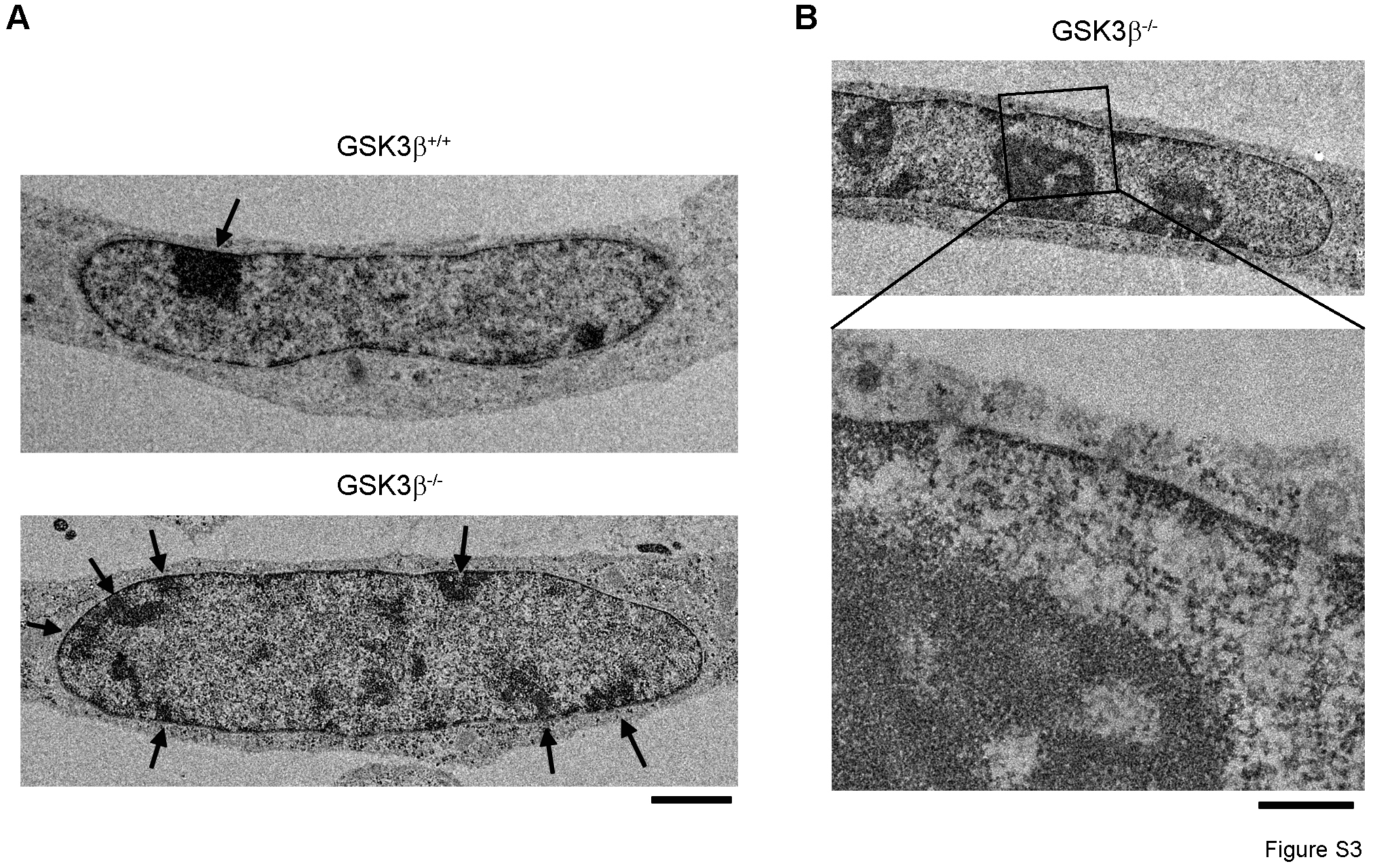

Supplement: Figure S3 — The ultrastructure of the nucleus in the GSK3β knockout MEFs. (A) Overview images showing the morphology of the cell nuclei of GSK3β+/+ and GSK3β−/− MEFs, as indicated. In GSK3β+/+ MEFs, the dense chromatin is typically concentrated in a few large domains (arrow), whereas the nucleus of GSK3β−/− MEFs contains a larger number of smaller chromatin patches (arrows) that are often associated with nucleolar material. The magnification bar represents 1 µm. (B) An example of GSK3β−/− MEFs displaying large and very vacuolated nucleoli. The magnification bar represents 200 nm. (TIF) [file pgen.1004390.s003.tif]

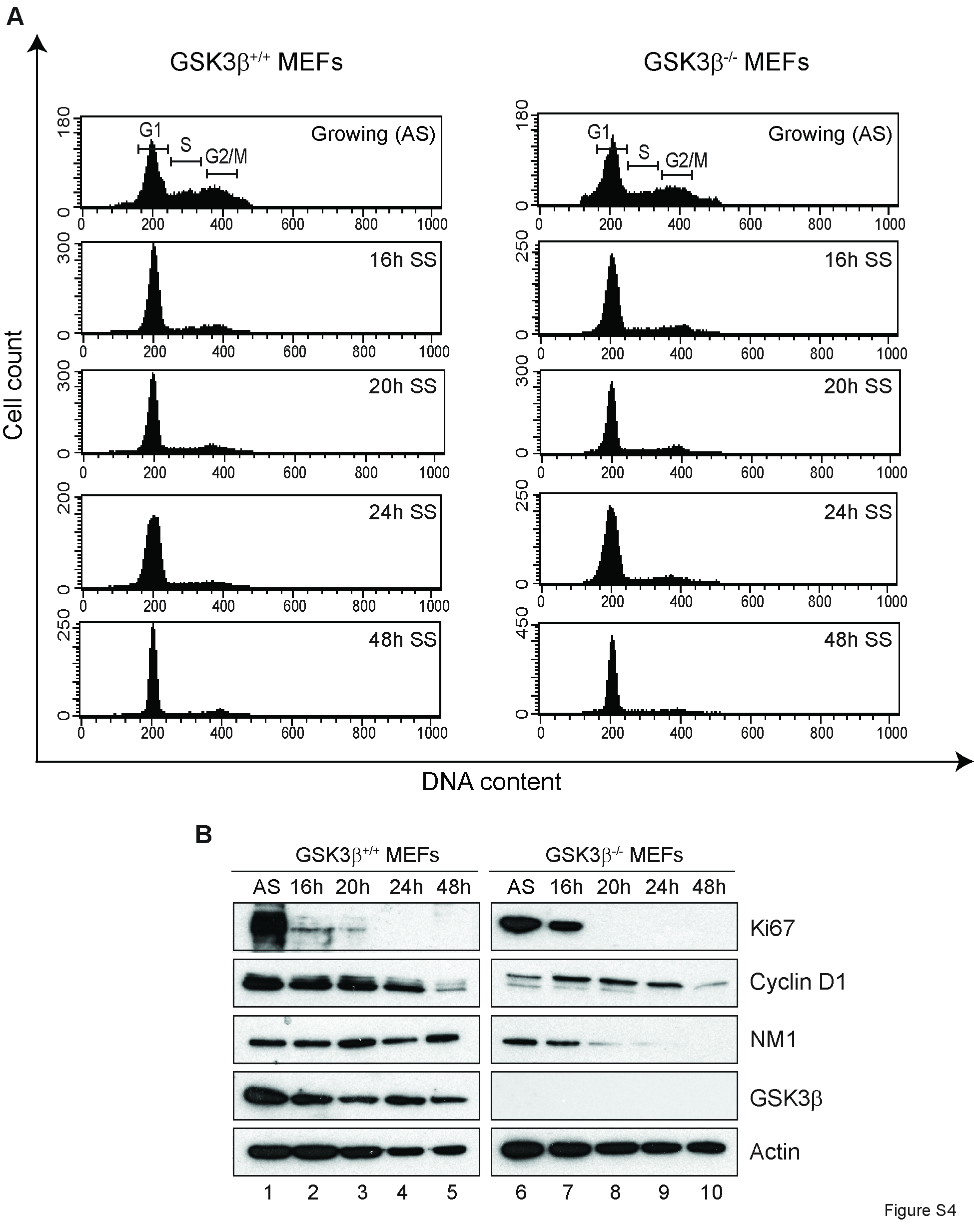

Supplement: Figure S4 — GSK3β+/+ MEFs and GSK3β−/− MEFs subjected to serum starvation. (A) Cell cycle profile for growing and time-course serum starvation (16 h, 20 h, 24 h, 48 h) performed on propidium iodide-stained GSK3β+/+ MEFs and GSK3β−/− MEFs by FACS. (B) Immunoblots of lysates obtained from growing and serum starved GSK3β+/+ MEFs and GSK3β−/− MEFs at the time points 16 h, 20 h, 24 h, 48 h, using antibodies to Ki67, cyclin D1, NM1, GSK3β and β-actin. (TIF) [file pgen.1004390.s004.tif]

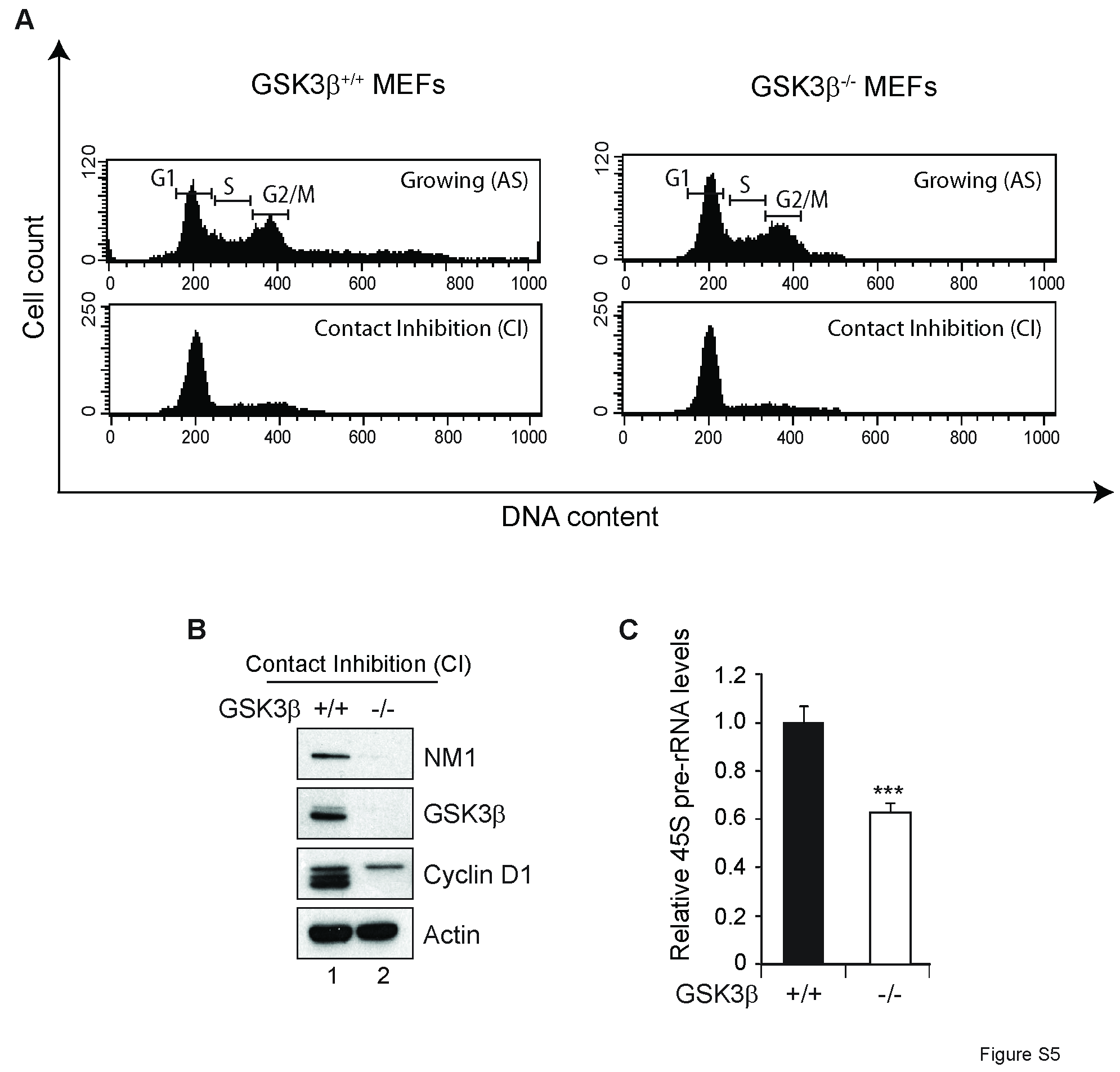

Supplement: Figure S5 — GSK3β+/+ MEFs and GSK3β−/− MEFs blocked in G1 by contact inhibition. (A) Cell cycle profile for growing and G1-arrested GSK3β+/+ MEFs and GSK3β−/− MEFs by FACS on propidium iodide-stained cells. (B) Immunoblots of lysates from GSK3β+/+ MEFs and GSK3β−/− MEFs arrested in G1 by contact inhibition using antibodies for NM1, cyclin D1, and β-actin. (C) rRNA synthesis in GSK3β+/+ MEFs and GSK3β−/− MEFs arrested in G1 by contact inhibition. For the analysis, relative 45S pre-rRNA levels were monitored from total RNA preparations by RT–qPCR using tubulin mRNA as internal control [p = 5.4e-05, ***]. (TIF) [file pgen.1004390.s005.tif]

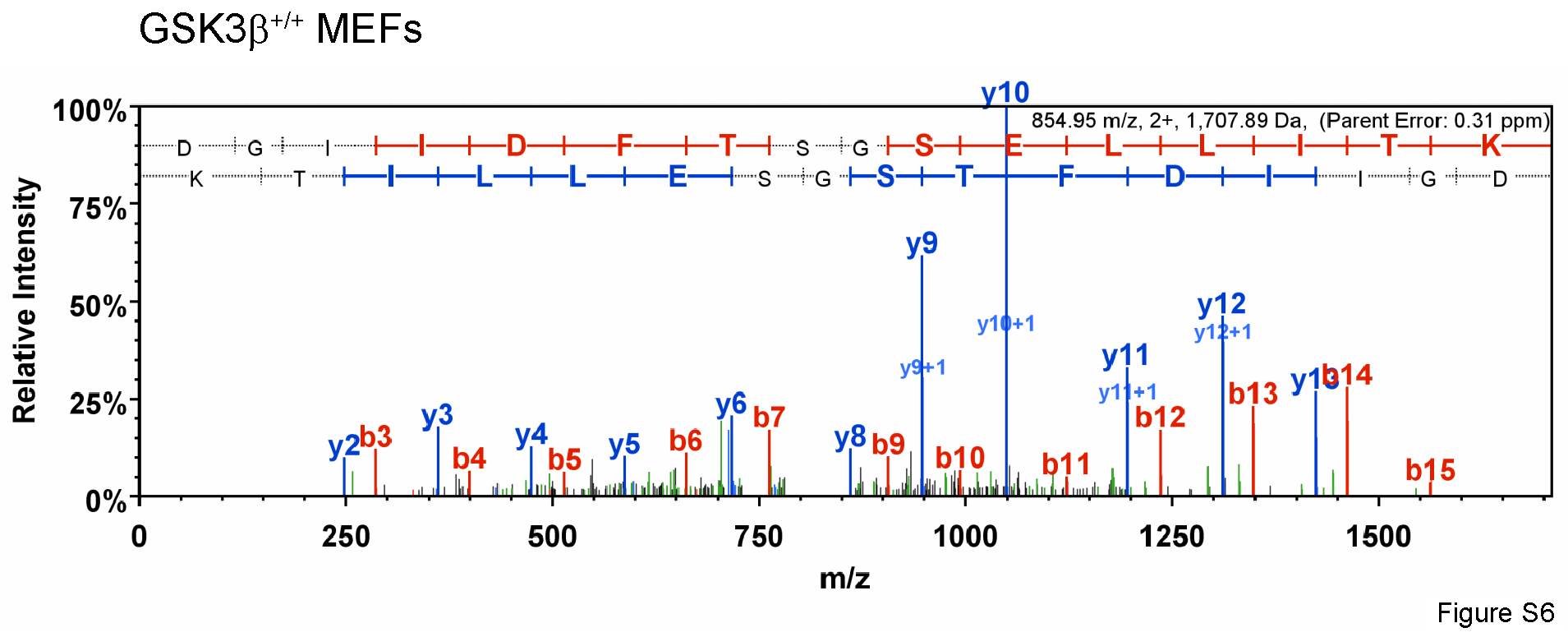

Supplement: Figure S6 — Tandem MS spectrum of non-phosphorylated peptide DGIIDFTSGSELLITK identified within the primary NM1 sequence immunoprecipitated from G1-arrested nuclear lysate of GSK3β+/+ MEFs. (TIF) [file pgen.1004390.s006.tif]

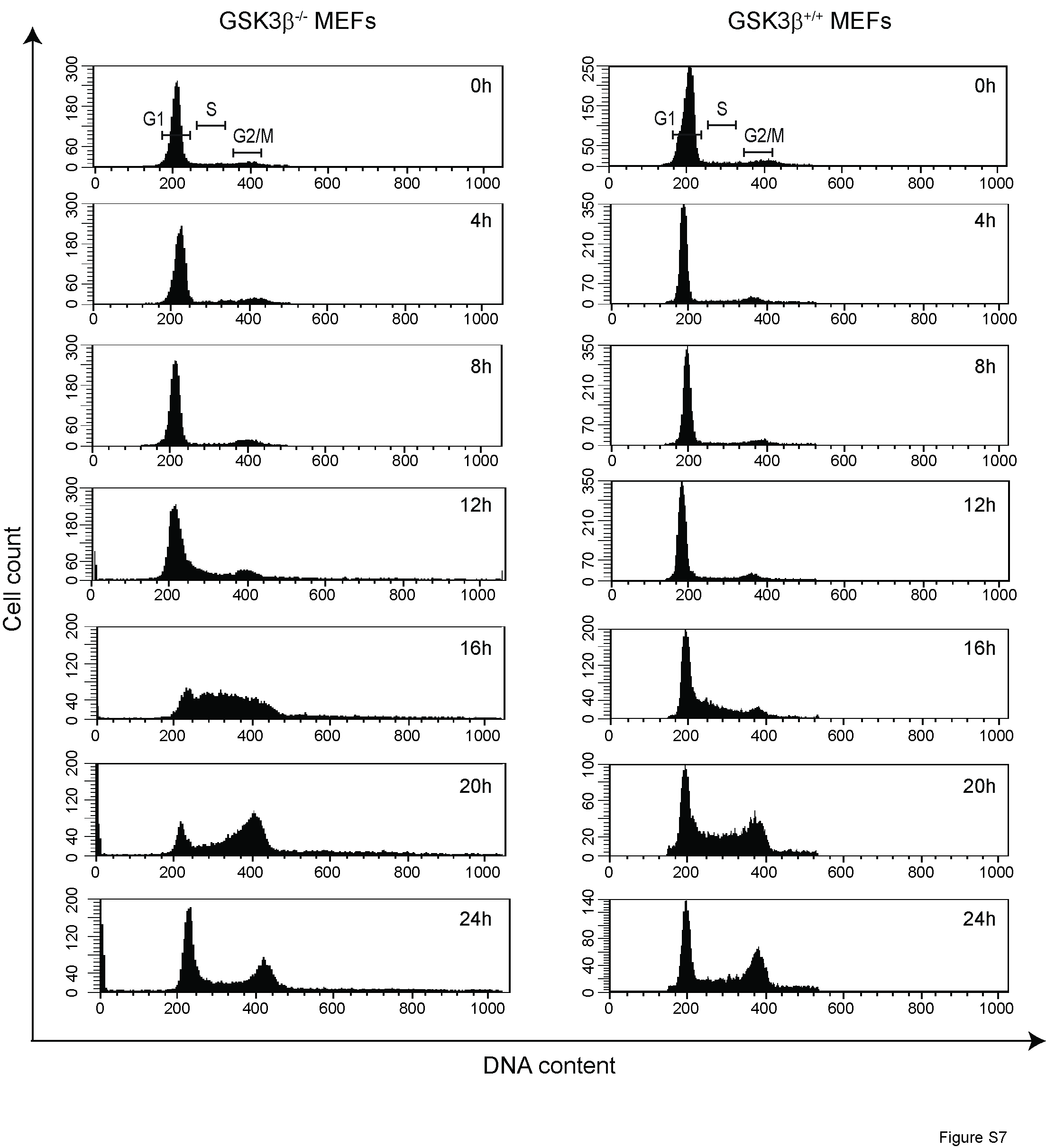

Supplement: Figure S7 — Cell cycle profile analyzed at the indicated time points after release from a G1 arrest by serum starvation using FACS on propidium iodide-stained GSK3β+/+ MEFs and GSK3β−/− MEFs. (TIF) [file pgen.1004390.s007.tif]

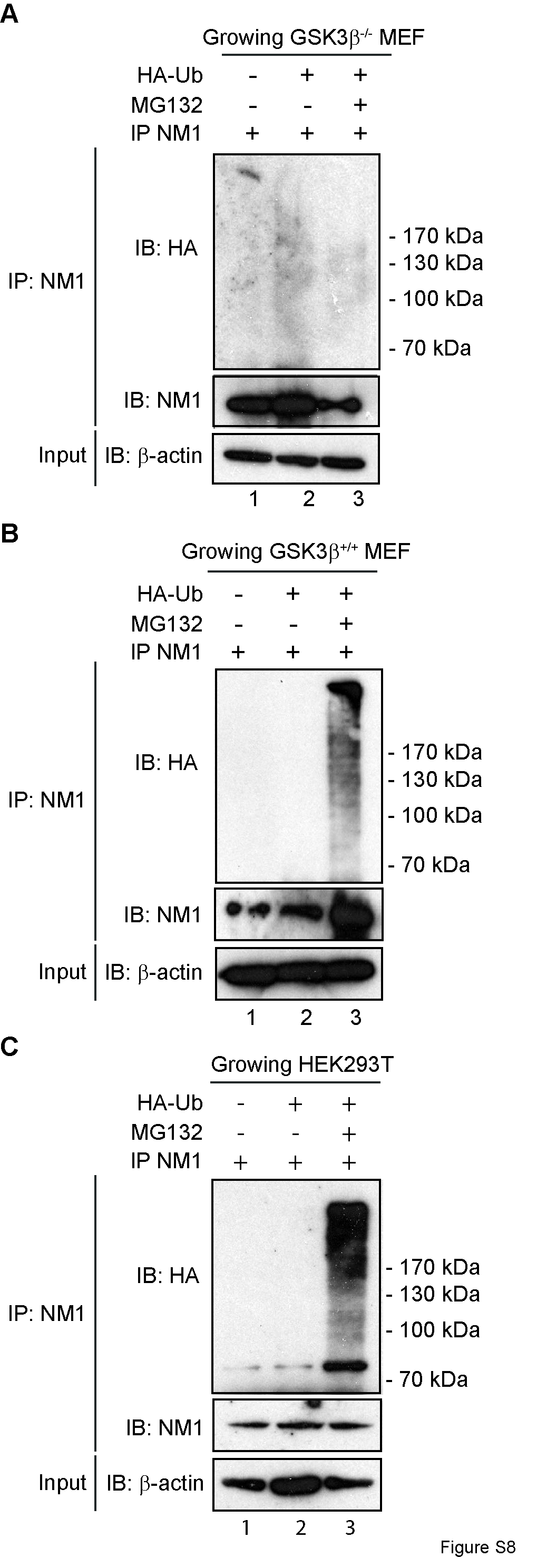

Supplement: Figure S8 — In growing cells NM1 is not ubiquitinated in a GSK3β-dependent manner. (A) Lysates were prepared from growing GSK3β−/− MEFs transiently expressing HA-tagged ubiquitin. The lysates were subjected to immunoprecipitations with the anti-NM1 antibody and the co-immunoprecipitated fractions were analyzed on immunoblots for HA-tagged ubiquitin. Lane 1, immunoprecipitationf from growing GSK3β−/− MEFs which do not express HA-tagged ubiquitin; lane 2, immunoprecipitations from untreated growing GSK3β−/− MEFs expressing HA-tagged ubiquitin; lane 3, immunoprecipitations from growing GSK3β−/− MEFs expressing HA-tagged ubiquitin treated with MG132. (B) Lysates were prepared from growing GSK3β+/+ MEFs transiently expressing HA-tagged ubiquitin. The lysates were subjected to immunoprecipitations with the anti-NM1 antibody and the co-immunoprecipitated fractions were analyzed on immunoblots for HA-tagged ubiquitin. Lane 1, immunoprecipitations from growing GSK3β+/+ MEFs which do not express HA-tagged ubiquitin; lane 2, immunoprecipitations from growing GSK3β+/+ MEFs expressing HA-tagged ubiquitin; lane 3, immunoprecipitations from growing GSK3β+/+ MEFs expressing HA-tagged ubiquitin treated with MG132. (C) Lysates were prepared from growing HEK293T cells transiently expressing HA-tagged ubiquitin. The lysates were subjected to immunoprecipitations with the anti-NM1 antibody and the co-immunoprecipitated fractions were analyzed on immunoblots for HA-tagged ubiquitin. Lane 1, immunoprecipitations from growing HEK293T cells which do not express HA-tagged ubiquitin; lane 2, immunoprecipitations from untreated growing HEK293T cells expressing HA-tagged ubiquitin; lane 3, immunoprecipitations from growing HEK293T cells expressing HA-tagged ubiquitin treated with MG132. (TIF) [file pgen.1004390.s008.tif]
