# Supplementary material for: Glycogen Synthase Kinase (GSK) 3β Phosphorylates and Protects Nuclear Myosin 1c from Proteasome-Mediated Degradation to Activate rDNA Transcription in Early G1 Cells
Source: PLoS Genet. 2014 Jun 5;10(6):e1004390. doi: 10.1371/journal.pgen.1004390 (PMC4046919; doi:10.1371/journal.pgen.1004390)
Supplement: Table S1 — GSK3β binding genome-wide. Mouse genomic regions corresponding to high levels of deep sequencing hits from the ChIP-Seq analysis. (DOC) [file pgen.1004390.s009.doc]

**Table S1.** GSK3β binding genome-wide. Mouse genomic regions corresponding to high levels of deep sequencing hits from the ChIP-Seq analysis.

| **Chromosome** | **Position** | **Gene** |
| --- | --- | --- |
| Chromosome 2 | 98502351 – 98504201 | [AK156724](http://www.ncbi.nlm.nih.gov/entrez/query.fcgi?cmd=Search&db=Nucleotide&term=AK156724&doptcmdl=GenBank&tool=genome.ucsc.edu) |
| Chromosome 4 | 70,039,099-70,039,357 | [Cdk5rap2](http://www.ncbi.nlm.nih.gov/gene?cmd=Retrieve&dopt=Graphics&list_uids=214444) |
| Chromosome 6 | 103,598,934-103,599,488 | [Chl1](http://www.ncbi.nlm.nih.gov/gene/12661) |
| Chromosome 9 | 3,013,025-3,041,236 | [mir101c](http://www.ncbi.nlm.nih.gov/gene/?term=mir101c) |
| Chromosome 9 | 35,112,747-35,113,244 | - |
| Chromosome 11 | 3,027,073-3,100,362 | [Sfi1](http://www.ncbi.nlm.nih.gov/gene/78887) |
| Chromosome 12 | 3,109,879-3,110,259 | - |
| Chromosome 17 | 39,979,760-39,986,816 | [RN45S](http://www.ncbi.nlm.nih.gov/gene/100861531) |
| Chromosome X | 73,843,955-73,844,788 | [NM_001201395.1](http://www.ncbi.nlm.nih.gov/entrez/query.fcgi?cmd=Search&db=Nucleotide&term=NM_001201395&doptcmdl=GenBank&tool=genome.ucsc.edu) |
| Chromosome X | 109,484,314-109,484,869 | [Apoo1](http://www.ncbi.nlm.nih.gov/nuccore/NM_026565?report=GenBank) |
| Chromosome X | 139,917,617-139,917,700 | - |
| Chromosome X | 166,403,937-166,444,158 | [Mid1](http://www.ncbi.nlm.nih.gov/gene/17318) |
