# Supplementary material for: Glycogen Synthase Kinase (GSK) 3β Phosphorylates and Protects Nuclear Myosin 1c from Proteasome-Mediated Degradation to Activate rDNA Transcription in Early G1 Cells
Source: PLoS Genet. 2014 Jun 5;10(6):e1004390. doi: 10.1371/journal.pgen.1004390 (PMC4046919; doi:10.1371/journal.pgen.1004390)
Supplement: Table S4 — Sequences of mouse and human oligonucleotide primers used in the qPCR and qRT PCR analyses. For primer sequences, see references [7], [9], [50]. (DOC) [file pgen.1004390.s012.doc]

**Table S4.** Sequences of mouse and human primers used in the qPCR and qRT PCR analyses.

| **Primer name** | **Sequence** | **Reference** |
| --- | --- | --- |
| NM1_forward  NM1_reverse | GGG CAG GAT GCG CTA CC  TGA GCG CAC TCT CCA TGG T | [7] Philimonenko et al., 2004 |
| Actin_forward  Actin_reverse | AGCCTTCCTTCCTGGGTA  CACTGTGTTGGCATAGAGGT | [9] Sarshad et al., 2013 |
| Tubulin_forward  Tubulin_reverse | AGCGCAGCATCCAGTTGT  CTGTGGTGTTGCTCAGCATAG | [9] Sarshad et al., 2013 |
| 45S_forward  45S_reverse | GCTTGTTTCTCCCGATTGC  CGCGAACCACTGAGAAAAGT | [50] Young et al, 2007 |
| 45S-2_forward  45S-2_reverse | TCCGATAGGGCTACACAGAAA  ATTTTAGGCACGACTTCCCC |  |
| 18S_forward  18S_reverse | CGACGACCCATTCGAACGTCT  CTCTCCGGAATCGAACCCTGA | [50] Young et al, 2007 |
| 28S_forward  28S_reverse | AGTCGGGTTGCTTGGGAATGC  CCCTTACGGTACTTGTTGACT | [50] Young et al, 2007 |
| 5.8S_forward  5.8S_reverse | AAG CGA CGC TCA GAC AGG  ACT CTT AGC GGT GGA TCA CTC | [50] Young et al., 2007 |
| IGS_forward  IGS_reverse | ATCAGGAGGTCCCGCTAGTT  ACGGCTTGACATCCAAACTC | [50] Young et al, 2007 |
| IGS-1_forward  IGS-1_reverse | ATCTTGTTGTGCGGGAGTTC  TTGTTCTGTCACTCGGTTGC |  |
| IGS-2_forward  IGS-2_reverse | TTAAAGACATGCGCCATCAC  GAGTTCCAGGTCAGCCAGAG |  |
